# Experimental Identification of the Second‐Order Non‐Hermitian Skin Effect with Physics‐Graph‐Informed Machine Learning

**DOI:** 10.1002/advs.202202922

**Published:** 2022-11-13

**Authors:** Ce Shang, Shuo Liu, Ruiwen Shao, Peng Han, Xiaoning Zang, Xiangliang Zhang, Khaled Nabil Salama, Wenlong Gao, Ching Hua Lee, Ronny Thomale, Aurélien Manchon, Shuang Zhang, Tie Jun Cui, Udo Schwingenschlögl

**Affiliations:** ^1^ King Abdullah University of Science and Technology (KAUST) Physical Science and Engineering Division (PSE) Thuwal 23955‐6900 Saudi Arabia; ^2^ State Key Laboratory of Millimeter Waves Southeast University Nanjing 210096 China; ^3^ King Abdullah University of Science and Technology (KAUST), Computer, Electrical and Mathematical Sciences and Engineering Division (CEMSE) Thuwal 23955‐6900 Saudi Arabia; ^4^ Department of Computer Science and Engineering University of Notre Dame Notre Dame IN 46556 USA; ^5^ Paderborn University Department of Physics Warburger Str. 100 33098 Paderborn Germany; ^6^ Department of Physics National University of Singapore Singapore 117551 Republic of Singapore; ^7^ Institut für Theoretische Physik und Astrophysik Universität Würzburg 97074 Würzburg Germany; ^8^ CINaM Aix‐Marseille University CNRS Marseille France; ^9^ Department of Physics The University of Hong Kong Hong Kong China

**Keywords:** graph visualization, machine learning, non‐Hermitian circuit, skin effect, topology

## Abstract

Topological phases of matter are conventionally characterized by the bulk‐boundary correspondence in Hermitian systems. The topological invariant of the bulk in *d* dimensions corresponds to the number of (*d* − 1)‐dimensional boundary states. By extension, higher‐order topological insulators reveal a bulk‐edge‐corner correspondence, such that *n*th order topological phases feature (*d* − *n*)‐dimensional boundary states. The advent of non‐Hermitian topological systems sheds new light on the emergence of the non‐Hermitian skin effect (NHSE) with an extensive number of boundary modes under open boundary conditions. Still, the higher‐order NHSE remains largely unexplored, particularly in the experiment. An unsupervised approach—physics‐graph‐informed machine learning (PGIML)—to enhance the data mining ability of machine learning with limited domain knowledge is introduced. Through PGIML, the second‐order NHSE in a 2D non‐Hermitian topoelectrical circuit is experimentally demonstrated. The admittance spectra of the circuit exhibit an extensive number of corner skin modes and extreme sensitivity of the spectral flow to the boundary conditions. The violation of the conventional bulk‐boundary correspondence in the second‐order NHSE implies that modification of the topological band theory is inevitable in higher dimensional non‐Hermitian systems.

## Graphical abstract

Physics‐graph‐informed machine learning (PGIML), formulated by integrating physical principles, graph visualization, and machine learning, enables the identification of the second‐order non‐Hermitian skin effect in topological circuits and thus opens new avenues for understanding the interplay with higher‐order topology. PGIML establishes a powerful methodology for the description of extremely complex systems.

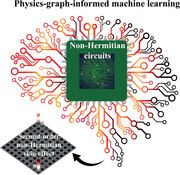

## 1 Introduction

Conceptual theories about topological phases of matter are at the forefront of contemporary research. In Hermitian systems, the guiding principle of topological insulators (TIs) is the bulk‐boundary correspondence, stating that the topological invariants of the bulk determine the number of gapless boundary modes.^[^ ^1^, ^2^, ^3^ ^]^ With progress in research, higher‐order TIs have revealed a novel bulk‐edge‐corner correspondence, where *n*th order topological phases in *d* dimensions feature (*d* − *n*)‐dimensional boundary modes.^[^ ^4^, ^5^, ^6^, ^7^, ^8^, ^9^, ^10^, ^11^, ^12^, ^13^, ^14^, ^15^, ^16^ ^]^ Building up on the categories of Hermitian systems, non‐conservative systems without Hermiticity reveal a plethora of unconventional physical principles, phenomena, and applications. Among many others, this includes parity‐time symmetry,^[^ ^17^, ^18^, ^19^ ^]^ exceptional points,^[^ ^20^ ^]^ exceptional Fermi arcs,^[^ ^21^ ^]^ sensing,^[^ ^22^, ^23^ ^]^ and lasing.^[^ ^24^, ^25^ ^]^ Recently, the concept of non‐Hermiticity has been intertwined with topological phases of matter^[^ ^26^, ^27^, ^28^, ^29^, ^30^, ^31^, ^32^, ^33^ ^]^ to yield the non‐Hermitian skin effect (NHSE) with an extensive number of boundary modes and the necessity to assess non‐Hermitian topological properties beyond Bloch band theory.^[^ ^34^, ^35^, ^36^ ^]^

Despite a fast‐growing number of theoretical predictions for non‐Hermitian topological systems,^[^ ^37^, ^38^, ^39^, ^40^, ^41^, ^42^, ^43^, ^44^, ^45^, ^46^, ^47^, ^48^, ^49^ ^]^ experimental explorations are still at an early stage.^[^ ^50^, ^51^, ^52^, ^53^, ^54^ ^]^ To date, the first‐order NHSE has been realized in photonic^[^ ^55^ ^]^ and in circuitry^[^ ^50^, ^51^, ^52^ ^]^ environments, whereas the experimental realization of the higher‐order NHSE remains open. Although skin corner modes have been observed in very recent research,^[^ ^53^, ^54^ ^]^ the unique features of the higher‐order NHSE need to be fully demonstrated, both the extensive number of boundary modes under open boundary conditions and the extreme sensitivity of the spectral flow to the boundary conditions. To analyze the spectral flow in higher dimensions, traditional methodologies are challenged by the large‐scale data generated. The data size will grow exponentially with the dimension, and additional boundary conditions make it more difficult to analyze the outcome. Machine learning (ML) is a promising way to process large amounts of data.^[^ ^56^, ^57^, ^58^ ^]^ The existing approaches, however, are unable to efficiently extract the crucial observables, in particular with a largely unexplored state of matter at hand. There is a pressing need for integrating fundamental physical laws and domain knowledge by teaching ML models the governing physical rules, which can, in turn, provide informative priors, that is, theoretical constraints and inductive understanding of the observable features. To this end, physics‐informed ML, using informative priors for the phenomenological description of the world, can be leveraged to improve the performance of the learning algorithm.^[^ ^59^ ^]^

In this article, we report two significant advances: i) The methodology of physics‐graph‐informed machine learning (PGIML) is introduced to enforce identification of an unrevealed physical phenomenon by integrating physical principles, graph visualization of features, and ML. The informative priors provided by PGIML enable an analysis that remains robust even in the presence of imperfect data (such as missing values, outliers, and noise) to make accurate and physically consistent predictions of phenomenological parameters. ii) The second‐order NHSE, characterized by skin corner modes and the violation of the conventional bulk‐boundary correspondence, is realized in a 2D non‐Hermitian topoelectrical circuit. We demonstrate experimentally the extreme sensitivity of the spectral flow to (fully controlled) boundary conditions (PBC*x*‐PBC*y*, PBC*x*‐OBC*y*, OBC*x*‐PBC*y*, and OBC*x*‐OBC*y*, where PBC (OBC) represents a periodic (open) boundary condition and *x* (*y*) represents direction), and observe corner skin modes under OBC*x*‐OBC*y* as well as edge skin modes under PBC*x*‐OBC*y*. Prospectively, the powerful tool of PGIML can be applied more widely to solve digital twin problems,^[^ ^60^, ^61^, ^62^ ^]^ thus bridging the physical and digital worlds by linking the flow of data/information between them.^[^ ^63^, ^64^ ^]^

## 2 Results

### 2.1 Physics‐Graph‐Informed Machine Learning

The PGIML framework is implemented in the context of a circuitry environment. In an electrical circuit, the scattering matrix (*S*‐matrix) relates the voltage of the waves incident to ports to those of the waves reflected from ports (see Section S1, Supporting Information), providing a complete description of the circuit.^[^ ^65^ ^]^ According to graph theory (network topology), an *N*‐port electrical circuit can be converted into a matrix ***G*** = (***P, S***) of complex‐weighted directed bipartite graphs $G_{ab}=\left(P_{ab},S_{ab}\right)$ with the matrix ***P*** of positions $P_{ab}=\left(a,b\right)$ and the *S*‐matrix ***S*** of scattering‐parameters (*S*‐parameters) *S*_*ab*_, where *a*, *b* ∈ {1, 2, …, *N*} denotes the ports.^[^ ^66^ ^]^ We define the set of graphs as $G=\left(P,S\right)=\{G_{ab}|a,b\in \left\{1,2,\cdots ,N\right\}\}$ with the set of positions P and the set of *S*‐parameters S. To identify the characteristic features of the circuit, especially of a large circuit, cluster analysis can be used to detect graphs with similar properties. Here, a *K*‐means clustering algorithm^[^ ^67^, ^68^ ^]^ is employed to partition G into *K* clusters $G_{\kappa }$ based on the value of the *S*‐parameter, where $G=\overset{K}{\underset{\kappa =1}{⋃}}G_{\kappa }$. The axiom of choice^[^ ^69^ ^]^ states that for every indexed $G_{\kappa }$ we can find a representative graph ${\hat{G}}_{\kappa }$ such that ${\hat{G}}_{\kappa }\in G_{\kappa }$. In a digital twin scenario of simulation and experiment, the set of simulated graphs $G_{\mathrm{sim}.}$ is generated to describe the numerical outcome that imitates the set of experimental graphs $G_{\exp.}$. As $G_{\mathrm{sim}.}$ and $G_{\exp.}$ are isomorphic, the subsets $G_{\mathrm{sim}.,\kappa }$ and $G_{\exp.,\kappa }$ are isomorphic.^[^ ^70^ ^]^ Therefore, PGIML can be understood in the teacher‐student scenario in the sense that the teacher ($G_{\mathrm{sim}.}$) imparts informative priors (${\hat{G}}_{\mathrm{sim}.}$) to the student ($G_{\exp.}$).

**Figure 1:**
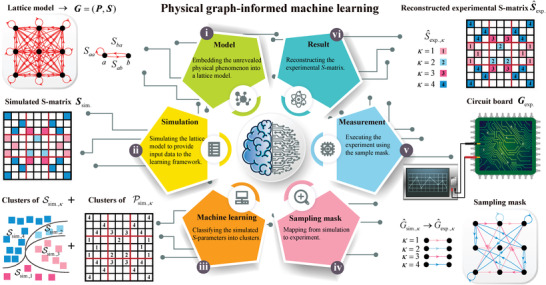
PGIML framework. A lattice model embedding the unrevealed physical phenomenon is generated. The directional red circles and lines correspond to $S_{aa}$ and $S_{ab}$, respectively (i). The simulated S‐matrix $S_{\mathrm{sim}}$. of the L×L lattice model is constructed and N×N ($N=L^{2}$) elements of a learning set $G_{\mathrm{sim}.}=\left(P_{\mathrm{sim}.},S_{\mathrm{sim}.}\right)$ are accumulated (ii). $P_{\mathrm{sim}.}$ and $S_{\mathrm{sim}.}$ are classified into clusters $P_{\mathrm{sim}.,\kappa }$ and $S_{\mathrm{sim}.,\kappa }$ using the K‐means method (iii). A sampling mask corresponding to the graph‐to‐graph mapping ${\hat{G}}_{\mathrm{sim}.,\kappa }\to {\hat{G}}_{\exp.,\kappa }$ is generated (iv). The representative experimental S‐parameters ${\hat{S}}_{\exp.,\kappa }$ are measured in the circuit (v). The reconstructed experimental S‐matrix ${\hat{S}}_{\exp.}$ is retrieved (vi).

We depict the PGIML framework in **Figure** **1**: i) A lattice model that embeds the unrevealed physical phenomenon is generated and converted into a matrix of graphs ***G***. ii) The simulated *S*‐matrix ***S***_sim._ of the circuit is constructed and a learning set $G_{\mathrm{sim}.}=\left(P_{\mathrm{sim}.},S_{\mathrm{sim}.}\right)$ is accumulated. iii) The set of simulated positions $P_{\mathrm{sim}.}$ and the set of simulated *S*‐parameters $S_{\mathrm{sim}.}$ are classified into clusters $P_{\mathrm{sim}.,\kappa }$ and $S_{\mathrm{sim}.,\kappa }$ using the *K*‐means method (see Section S3, Supporting Information). iv) The graph‐to‐graph mapping ${\hat{G}}_{\mathrm{sim}.,\kappa }\to {\hat{G}}_{\exp.,\kappa }$ is translated into a sampling mask that mirrors the clustering information. v) The representative experimental *S*‐parameters ${\hat{S}}_{\exp.,\kappa }$ are measured in the circuit. vi) The *S*‐matrix is encoded with the measured features $\overset{K}{\underset{\kappa =1}{⋃}}\left\{{\hat{S}}_{\exp.,\kappa }\right\}$ and the reconstructed experimental *S*‐matrix ${\hat{S}}_{\exp.}$ is retrieved. The experimental *S*‐matrix ***S***_exp._ is then given by

(1) $$\begin{array}{c} S_{\exp.}∼{\hat{S}}_{\exp.}=\sum_{\kappa =1}^{K}{\hat{S}}_{\exp.,\kappa }\sum_{\left(a,b\right)\in P_{\mathrm{sim}.,\kappa }}E_{ab}, \end{array}$$

where $E_{ab}$ is a single‐entry matrix (element *ab* is one and the other elements are zero).^[^ ^71^ ^]^ Compared to conventional measurements of *N* ^2^ elements, the PGIML method is *N* ^2^/*K* times faster, as it filters out redundancies, especially efficient for circuits that are too complex for a human to process.

### 2.2 Second‐Order Non‐Hermitian Skin Effect

**Figure 2:**
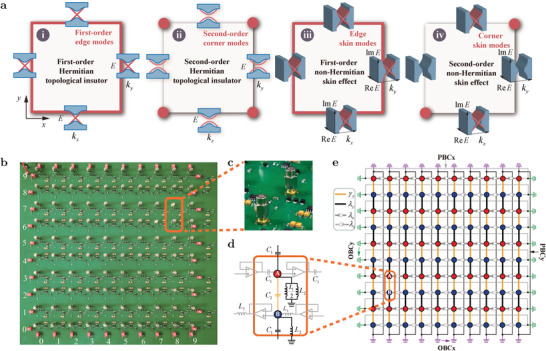
Second‐order NHSE in a non‐Hermitian circuit. a) Schematic diagrams of topological band theory for the first‐order TI, second‐order TI, first‐order NHSE, and second‐order NHSE. b) Photograph of the circuit. c) Photograph of the unit cell. d) Scheme of the unit cell. e) Tight‐binding analog of the circuit. The circuit components are represented by the intracell coupling $\gamma_{y}\;\left(C_{2}\right)$, intercell coupling $\lambda_{y}\;\left(C_{1}\right)$, and intercell non‐reciprocal couplings $\lambda_{x}$ ($C_{1}$ connected to a voltage follower) and $-\lambda_{x}$ ($L_{1}$ reversely connected to a voltage follower). The grounding components are $L_{1}L_{2}/\left(L_{1}+2L_{2}\right)$ and *alicmathL*_2_ for sublattices A and B, respectively. PBC*x* (gray), PBC*y* (black), OBC*x* (green), and OBC*y* (purple) are controlled by the switches connecting the boundaries (see Section S4, Supporting Information).

We are now set up to explore the second‐order NHSE, which gives rise to new types of boundary modes as a result of higher‐order non‐Hermitian topology. In a *L* × *L* lattice model, a first‐order TI has O(L) edge modes with a gapless edge spectrum in the *x*‐ and *y*‐directions. A second‐order TI has O(1) corner modes with a gapped edge spectrum in the *x*‐ and *y*‐directions. The first‐order NHSE features extensive $O(L^{2})$ edge skin modes with a gapless complex‐valued edge spectrum in the *x*‐ and *y*‐directions. Distinct from the Hermitian limit and the first‐order NHSE, the second‐order NHSE features O(L) corner skin modes with a gapless complex‐valued edge spectrum in one direction and no edge spectrum in the other direction (see Section S2, Supporting Information). Schematic diagrams of these four situations are shown in **Figure** **2**a. The explicit violation of the conventional bulk‐boundary correspondence clearly demonstrates that modification of the topological band theory is inevitable in higher‐dimensional non‐Hermitian systems.

To realize the second‐order NHSE experimentally, we design a topoelectrical circuit that represents a 2D non‐Hermitian two‐band model. The 10 × 10 circuitry lattice is shown in Figure 2b and the unit cell is shown in Figure 2c as photograph and in Figure 2d as scheme. The tight‐binding analog of the circuit is shown in Figure 2e with intracell couplings γ_*y*_, intercell couplings λ_*y*_ in the *y*‐direction, and intercell non‐reciprocal couplings ±λ_*x*_ in the *x*‐direction.

According to Kirchhoff's laws, any circuit can be described by the block diagonal admittance matrix (circuit Laplacian) $J\left(\omega \right)=i\omega C+\frac{1}{i\omega }W$, where ***C*** and ***W*** are the Laplacian matrices of the capacitance and inverse inductance, respectively. For a given input current of frequency ω = 2π*f*, we obtain the non‐reciprocal two‐band admittance matrix (see Section S1, Supporting Information)

(2) $$\;J\left(k,\omega \right)=i\omega \left[\begin{array}{cc} \frac{L_{1}L_{2}}{\left(L_{1}+2L_{2}\right)\omega^{2}}-2C_{1}-C_{2}+C_{1}e^{-ik_{x}} & C_{2}+C_{1}e^{-ik_{y}}\\ C_{2}+C_{1}e^{ik_{y}} & \frac{L_{1}L_{2}}{\left(L_{1}+L_{2}\right)\omega^{2}}-C_{1}-C_{2}-\frac{L_{1}}{\omega^{2}}e^{ik_{x}} \end{array}\right]$$

where two pairs of capacitors and inductors, (*C*_1_, *L*_1_) and (*C*_2_, *L*_2_), with the same resonance frequency $\omega_{0}=1/\sqrt{L_{1}C_{1}}=1/\sqrt{L_{2}C_{2}}$ are used to couple the nodes. This implies

(3) $$\begin{array}{ccc} J(k,\omega_{0}) & = & i\sqrt{C_{1}L_{1}}\left[-i\lambda_{x}\sin k_{x}\sigma_{0}+\lambda_{x}\cos k_{x}\sigma_{z}+\lambda_{y}\sin k_{y}\sigma_{y}\right.\\ & & \left.+\left(\gamma_{y}+\lambda_{y}\cos k_{y}\right)\sigma_{x}\right] \end{array}$$

For $C_{1}=1000\mathrm{pH}$, $C_{2}=330\mathrm{pH}$, $L_{1}=33\mu F$, and $L_{2}=100\mu F$, we arrive at λ_*x*_ = 1, λ_*y*_ = 1, and γ_*y*_ = 0.33. The eigenvalues of ***J*** (***k***, ω_0_) are given by

(4) $$\begin{array}{ccc} & & j(k,\omega_{0})\\ & & =i\sqrt{C_{1}/L_{1}}\left(\pm \sqrt{{\lambda_{x}}^{2}\cos^{2}k_{x}+2\lambda_{y}\gamma_{y}\cos k_{y}+{\lambda_{y}}^{2}+\gamma_{y}^{2}}-i\lambda_{x}\sin k_{x}\right) \end{array}$$

**Figure 3:**
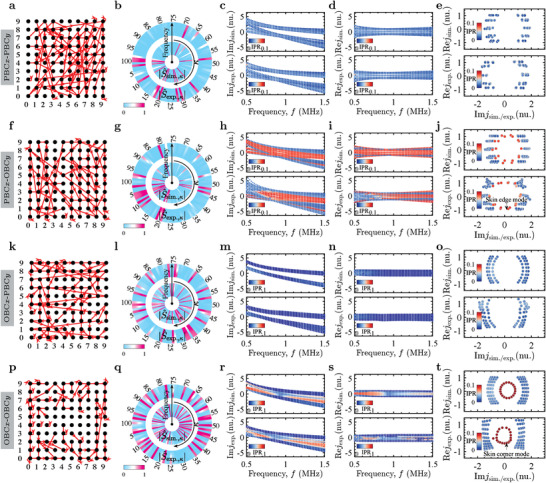
Comparison of experimental and simulated results for different boundary conditions. a,f,k,p) Representative S‐parameters are measured between ports connected by red directional circles and lines for κ=1,2,…,100. b,g,l,q) Frequency response of $|{\hat{S}}_{\mathrm{sim}.,\kappa }|$ (inner circle) and $|{\hat{S}}_{\exp.,\kappa }|$ (outer circle) for each κ, showing excellent agreement. c,d,h,i,m,n,r,s) Imaginary and real parts of the admittance spectra $j_{\mathrm{sim}}$. (top panel) and *j*_exp_. (bottom panel) as functions of the driving frequency f, weighted by the IPR. e,j,o,t) Complex admittance spectra for the resonance frequency $f_{0}\approx 0.876\;\mathrm{MHz}$.

**Figure 4:**
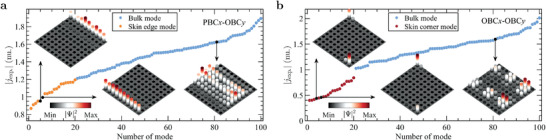
Admittance spectra and mode distributions. a) Absolute values of $j_{\exp.}$ for PBCx-OBCy. b) Absolute values of $j_{\exp.}$ for OBCx-OBCy. The insets show the bulk and skin edge/corner modes.

As the boundary connections can be customized, we can observe phase transitions through differences in the spectral flow, enabling the study of the topological modes at any choice of boundary conditions. The admittance eigenvalues and eigenstates are accessible by an *S*‐parameter measurement using the PGIML framework. We address the circuit for PBC*x*‐PBC*y* in **Figure** **3**a–e, for PBC*x*‐OBC*y* in Figure 3f–j, for OBC*x*‐PBC*y* in Figure 3k–o, and for PBC*x*‐PBC*y* in Figure 3p–t. Figure 3a,f,k,p shows the ports selected for measuring the representative *S*‐parameters (see Section S4, Supporting Information). According to Figure 3b,g,l,q, the frequency response of $|{\hat{S}}_{\exp.,\kappa }|$ (outer circle) agrees well with that of $|{\hat{S}}_{\mathrm{sim}.,\kappa }|$ (inner circle). Figures 3c,h,m,r and 3d,i,n,s show the imaginary and real parts, respectively, of the simulated (top panel) and experimental (bottom panel) admittance spectra as functions of the driving frequency *f*, weighted by the inverse participation ratio $\mathrm{IPR}=\sum_{n}{|\Psi_{n}|}^{4}/{\left(\sum_{n}{|\Psi_{n}|}^{2}\right)}^{2}$, where Ψ_*n*_ is the *n*th eigenmode. A larger IPR corresponds to a more localized mode. For simplicity, the results are given in normalized units (nu) as multiplies of $\sqrt{L_{1}/C_{1}}\;\Omega^{-1}$. Figure 3e,j,o,t shows the simulated and experimental admittance spectra in the complex plane at the resonance frequency $f_{0}={\left(2\pi \sqrt{L_{1}C_{1}}\right)}^{-1}\approx \;0.876\mathrm{MHz}$. The system has trivial topology without NHSE for PBC*x*‐PBC*y* and OBC*x*‐PBC*y*, and non‐trivial topology with NHSE for PBC*x*‐OBC*y* and OBC*x*‐OBC*y*. In particular, Figure 3j shows skin edge modes for PBC*x*‐OBC*y*. We observe in **Figure** **4**a a localized mode distribution at the left/right boundary, in contrast to the delocalized bulk modes. Remarkably, the skin corner modes in Figure 3t form a circle in the complex‐energy plane for OBC*x*‐OBC*y*, analytically given by $j_{\mathrm{sim}.}=0.33\sqrt{C_{1}/L_{1}}e^{i\theta },\theta \in \left[0,2\pi \right]$ (see Section S2, Supporting Information). They are localized at the corners while the bulk modes are delocalized, as can be seen in Figure 4b. A non‐Bloch 2D winding number *v*_2*D*_ = 1 characterizes the higher‐order NHSE (Experimental Section). For all the *L* ^2^ eigenmodes, the number of corner skin modes is 2*L* while the number of delocalized bulk modes is *L* ^2^ − 2*L*.

## 3 Conclusion

In times of digital research and measurement, many scientific disciplines produce large amounts of data that by far surpass conventional computational abilities for processing and analyzing. Hence, we develop the PGIML method by integrating physical principles, graph visualization of features, and ML to enforce the identification of an unrevealed physical phenomenon. At the example of a topoelectrical circuit, we embed the physical principles of the second‐order NHSE into the circuit, observe the skin corner modes, demonstrate the violation of the conventional bulk‐boundary correspondence, and reveal an intriguing interplay between higher‐order topology and non‐Hermiticity. Our results suggest that the PGIML method provides a paradigm shift in processing and analyzing data, opening new avenues to understanding complex systems in higher dimensions.

## 4 Experimental Section

### Topological invariant

According to point‐gap topology,^[^ ^35^, ^45^, ^72^ ^]^ a topological characterization of the NHSE was derived. A non‐Hermitian Hamiltonian *H* had a point gap at a reference point E∈C if and only if its complex spectrum does not cross *E*, that is, det(H−E)≠0. The topological invariant is given by the winding number

(5) $$w\left(E\right)=\int_{0}^{2\pi }\frac{dk}{2\pi i}\frac{d}{dk}\log\det\left[H\left(k\right)-E\right]$$

where H(k) is the non‐Hermitian Bloch Hamiltonian. The second‐order NHSE occurred when w(E)≠0. The non‐Hermitian topology of H(k) could also be understood in terms of the extended Hermitian Hamiltonian

(6) $$\tilde{H}\left(k,E\right)=\left(\begin{array}{cc} 0 & H(k)-E\\ H^{†}\left(k\right)-E^{*} & 0 \end{array}\right)$$

which is topologically nontrivial with a finite energy gap if and only if H(k) is topologically nontrivial with a point gap at *E*.

To clarify the topological property of the second‐order NHSE,^[^ ^73^, ^74^, ^75^ ^]^ the extended Hermitian admittance Hamiltonian was defined as

(7) $$\tilde{J}\left(k,\omega_{0}\right)=\left(\begin{array}{cc} 0 & J(k,\omega_{0})-j\\ J^{†}\left(k,\omega_{0}\right)-j^{*} & 0 \end{array}\right)$$

and the unitary transformation $\tilde{J}\left(k,\omega_{0}\right)=U\tilde{J}\left(k,\omega_{0}\right)U^{†}$ was performed using

(8) $$U=\left(\begin{array}{cccc} 0 & 0 & 0 & -1\\ 1 & 0 & 0 & 0\\ 0 & -1 & 0 & 0\\ 0 & 0 & 1 & 0 \end{array}\right)$$

and obtained

(9) $$\tilde{J}\left(k,\omega_{0}\right)={\tilde{J}}_{x}\left(k_{x},\omega_{0}\right)⊗\tau_{z}+\sigma_{0}⊗{\tilde{J}}_{y}\left(k_{y},\omega_{0}\right)$$

with

(10) $$\begin{array}{c} {\tilde{J}}_{x}\left(k_{x},\omega_{0}\right)=-i\sqrt{C_{1}L_{1}}\left[\lambda_{x}\cos k_{x}\sigma_{x}+\left(\lambda_{x}\sin k_{x}-E\right)\sigma_{y}\right]\\ {\tilde{J}}_{y}\left(k_{y},\omega_{0}\right)=-i\sqrt{C_{1}L_{1}}\left[\left(\lambda_{y}\cos k_{y}+\gamma_{y}\right)\tau_{x}-\lambda_{y}\sin k_{y}\tau_{y}\right] \end{array}$$

Both ${\tilde{J}}_{x}\left(k_{x},\omega_{0}\right)$ and ${\tilde{J}}_{y}\left(k_{x},\omega_{0}\right)$ had chiral symmetry corresponding to $\sigma_{z}$ and $\tau_{z}$, respectively. Since chirality and inversion symmetry here commute, the non‐Hermitian topology of $\tilde{J}\left(k,\omega_{0}\right)$ was characterized by the chiral symmetry $C=\sigma_{z}⊗\tau_{z}$. Thus, the second‐order NHSE was characterized by the Z topological invariant^[^ ^44^, ^75^ ^]^

(11) $$v_{2D}=w_{x}w_{y}$$

with the winding numbers

(12) $$w_{\alpha }\left(j\right)=\int_{0}^{2\pi }\frac{dk_{\alpha }}{2\pi i}\frac{d}{dk_{\alpha }}\log\det\left[{\tilde{J}}_{\alpha }\left(k_{\alpha },\omega_{0}\right)-j\right]$$

where α=x,y. Thus, $w_{x}=1$ as $E\in (-\;\lambda_{x},\lambda_{x})$ and $w_{y}=1$ as $\lambda_{y}/\gamma_{y}>1$. Hence, a nonzero topological invariant $v_{2D}=1$ was obtained if and only if $E\in (-\;\lambda_{x},\lambda_{x})$ and $\lambda_{y}/\gamma_{y}>1$. $v_{2D}$ changed when the edge and bulk modes close the gap, establishing the second‐order non‐Hermitian topology.

### Experiment

Nonreciprocal couplings were realized by voltage feedback operational amplifiers (Texas Instruments, LM6171), which blocked the input current while maintaining the output current. To ensure small linewidths of the circuit Laplacian spectra, high‐*Q* inductors (Murata, *Q*‐factor >40 with 5% component variation) were used. Additional elements were added to the circuit to increase the stability of the voltage feedback operational amplifiers, including a 5Ω resistor connected in series at the output and a 2000Ω resistor in shunt with a 100pF capacitor connecting across the inverting input and output of the voltage feedback operational amplifier. The circuit Laplacian spectra were obtained by measuring the S‐parameters of the circuit at 10kHz frequency resolution. A vector network analyzer (Tektronix TTr500) was employed and the S‐matrix was transformed into the circuit Laplacian using the impedance matrix, that is, the inverse of the circuit Laplacian $J^{-1}=Z_{0}\left(S+I\right){\left(I-S\right)}^{-1}$, where I is the identity matrix and *Z*_0_ is the characteristic impedance. In an S‐parameter measurement between two ports, the other ports were connected with 50Ω load terminators to ensure zero reflection. Note that the impedance matrix obtained by this method was equivalent to that obtained by current probes,^[^ ^76^, ^77^ ^]^ while the measurement was simplified dramatically and the experimental stability was improved.

## Conflict of Interest

The authors declare no conflict of interest.

## Author Contributions

C.S., S.L., and R.S. contributed equally to this work. C.S. and S.L. conceived the idea. C.S. performed the theoretical analyses. C.S., S.L., and R.S. designed the circuits and performed the experiments. C.S. and P.H. developed the machine learning method. C.H.L. and R.T. evaluated the experimental and numerical results. A.M., S.Z., T.J.C., and U.S. guided the research. All the authors contributed to the discussions of the results and the preparation of the manuscript.

## Supporting information

Supporting Information

## Data Availability

The data that support the findings of this study are available from the corresponding author upon reasonable request.

## References

[1] M. Z. Hasan , C. L. Kane , Rev. Mod. Phys. 2010, 82, 3045.

[2] X.‐L. Qi , S.‐C. Zhang , Rev. Mod. Phys. 2011, 83, 1057.

[3] A. Bansil , H. Lin , T. Das , Rev. Mod. Phys. 2016, 88, 021004.

[4] P. Sessi , D. D. Sante , A. Szczerbakow , F. Glott , S. Wilfert , H. Schmidt , T. Bathon , P. Dziawa , M. Greiter , T. Neupert , G. Sangiovanni , T. Story , R. Thomale , M. Bode , Science 2016, 354, 1269. 27940869 10.1126/science.aah6233

[5] W. A. Benalcazar , B. A. Bernevig , T. L. Hughes , Science 2017, 357, 61. 28684520 10.1126/science.aah6442

[6] W. A. Benalcazar , B. A. Bernevig , T. L. Hughes , Phys. Rev. B 2017, 96, 245115.

[7] Y. Peng , Y. Bao , F. von Oppen , Phys. Rev. B 2017, 95, 235143.

[8] Z. Song , Z. Fang , C. Fang , Phys. Rev. Lett. 2017, 119, 246402. 29286745 10.1103/PhysRevLett.119.246402

[9] J. Langbehn , Y. Peng , L. Trifunovic , F. von Oppen , P. W. Brouwer , Phys. Rev. Lett. 2017, 119, 246401. 29286744 10.1103/PhysRevLett.119.246401

[10] F. Schindler , A. M. Cook , M. G. Vergniory , Z. Wang , S. S. P. Parkin , B. A. Bernevig , T. Neupert , Sci. Adv. 2018, 4, eaat0346. 29869644 10.1126/sciadv.aat0346 PMC5983919

[11] M. Ezawa , Phys. Rev. B 2018, 97, 155305.

[12] X.‐L. Sheng , C. Chen , H. Liu , Z. Chen , Z.‐M. Yu , Y. X. Zhao , S. A. Yang , Phys. Rev. Lett. 2019, 123, 256402. 31922761 10.1103/PhysRevLett.123.256402

[13] M. J. Park , Y. Kim , G. Y. Cho , S. Lee , Phys. Rev. Lett. 2019, 123, 216803. 31809156 10.1103/PhysRevLett.123.216803

[14] R. Chen , C.‐Z. Chen , J.‐H. Gao , B. Zhou , D.‐H. Xu , Phys. Rev. Lett. 2020, 124, 036803. 32031860 10.1103/PhysRevLett.124.036803

[15] B. Huang , W. V. Liu , Phys. Rev. Lett. 2020, 124, 216601. 32530681 10.1103/PhysRevLett.124.216601

[16] Y. Ren , Z. Qiao , Q. Niu , Phys. Rev. Lett. 2020, 124, 166804. 32383951 10.1103/PhysRevLett.124.166804

[17] C. E. Rüter , K. G. Makris , R. El‐Ganainy , D. N. Christodoulides , M. Segev , D. Kip , Nat. Phys. 2010, 6, 192.

[18] A. Regensburger , C. Bersch , M.‐A. Miri , G. Onishchukov , D. N. Christodoulides , U. Peschel , Nature 2012, 488, 167. 22874962 10.1038/nature11298

[19] B. Peng , Ş. K. Özdemir , F. Lei , F. Monifi , M. Gianfreda , G. L. Long , S. Fan , F. Nori , C. M. Bender , L. Yang , Nat. Phys. 2014, 10, 394.

[20] J. Zhang , B. Peng , Ş. K. Özdemir , K. Pichler , D. O. Krimer , G. Zhao , F. Nori , Y.‐X. Liu , S. Rotter , L. Yang , Nat. Photonics 2018, 12, 479.

[21] H. Zhou , C. Peng , Y. Yoon , C. W. Hsu , K. A. Nelson , L. Fu , J. D. Joannopoulos , M. Soljačić , B. Zhen , Science 2018, 359, 1009. 29326118 10.1126/science.aap9859

[22] H. Hodaei , A. U. Hassan , S. Wittek , H. Garcia‐Gracia , R. El‐Ganainy , D. N. Christodoulides , M. Khajavikhan , Nature 2017, 548, 187. 28796201 10.1038/nature23280

[23] W. Chen , Ş. K. Özdemir , G. Zhao , J. Wiersig , L. Yang , Nature 2017, 548, 192. 28796206 10.1038/nature23281

[24] H. Hodaei , M.‐A. Miri , M. Heinrich , D. N. Christodoulides , M. Khajavikhan , Science 2014, 346, 975. 25414308 10.1126/science.1258480

[25] M. Brandstetter , M. Liertzer , C. Deutsch , P. Klang , J. Schöberl , H. E. Türeci , G. Strasser , K. Unterrainer , S. Rotter , Nat. Commun. 2014, 5, 4034. 24925314 10.1038/ncomms5034 PMC4082637

[26] S. Weimann , M. Kremer , Y. Plotnik , Y. Lumer , S. Nolte , K. G. Makris , M. Segev , M. C. Rechtsman , A. Szameit , Nat. Mater. 2017, 16, 433. 27918567 10.1038/nmat4811

[27] B. Bahari , A. Ndao , F. Vallini , A. E. Amili , Y. Fainman , B. Kanté , Science 2017, 358, 636. 29025992 10.1126/science.aao4551

[28] M. A. Bandres , S. Wittek , G. Harari , M. Parto , J. Ren , M. Segev , D. N. Christodoulides , M. Khajavikhan , Science 2018, 359, eaar4005. 29420263 10.1126/science.aar4005

[29] G. Harari , M. A. Bandres , Y. Lumer , M. C. Rechtsman , Y. D. Chong , M. Khajavikhan , D. N. Christodoulides , M. Segev , Science 2018, 359, eaar4003. 29420260 10.1126/science.aar4003

[30] L. Xiao , T. Deng , K. Wang , G. Zhu , Z. Wang , W. Yi , P. Xue , Nat. Phys. 2020, 16, 761.

[31] W. Zhang , X. Ouyang , X. Huang , X. Wang , H. Zhang , Y. Yu , X. Chang , Y. Liu , D.‐L. Deng , L.‐M. Duan , Phys. Rev. Lett. 2021, 127, 090501. 34506190 10.1103/PhysRevLett.127.090501

[32] L.‐W. Yu , D.‐L. Deng , Phys. Rev. Lett. 2021, 126, 240402. 34213933 10.1103/PhysRevLett.126.240402

[33] K. Zhang , Z. Yang , C. Fang , Nat. Commun. 2022, 13, 2496. 35523795 10.1038/s41467-022-30161-6 PMC9076925

[34] F. K. Kunst , E. Edvardsson , J. C. Budich , E. J. Bergholtz , Phys. Rev. Lett. 2018, 121, 026808. 30085697 10.1103/PhysRevLett.121.026808

[35] K. Kawabata , K. Shiozaki , M. Ueda , M. Sato , Phys. Rev. X 2019, 9, 041015.

[36] K. Yokomizo , S. Murakami , Phys. Rev. Lett. 2019, 123, 066404. 31491170 10.1103/PhysRevLett.123.066404

[37] S. Yao , Z. Wang , Phys. Rev. Lett. 2018, 121, 086803. 30192628 10.1103/PhysRevLett.121.086803

[38] F. Song , S. Yao , Z. Wang , Phys. Rev. Lett. 2019, 123, 170401. 31702238 10.1103/PhysRevLett.123.170401

[39] X.‐W. Luo , C. Zhang , Phys. Rev. Lett. 2019, 123, 073601. 31491088 10.1103/PhysRevLett.123.073601

[40] C. H. Lee , L. Li , J. Gong , Phys. Rev. Lett. 2019, 123, 016805. 31386404 10.1103/PhysRevLett.123.016805

[41] K. Zhang , Z. Yang , C. Fang , Phys. Rev. Lett. 2020, 125, 126402. 33016766 10.1103/PhysRevLett.125.126402

[42] Z. Yang , K. Zhang , C. Fang , J. Hu , Phys. Rev. Lett. 2020, 125, 226402. 33315431 10.1103/PhysRevLett.125.226402

[43] K. Kawabata , M. Sato , K. Shiozaki , Phys. Rev. B 2020, 102, 205118. 10.1103/PhysRevLett.124.086801 32167324

[44] R. Okugawa , R. Takahashi , K. Yokomizo , Phys. Rev. B 2020, 102, 241202.

[45] N. Okuma , K. Kawabata , K. Shiozaki , M. Sato , Phys. Rev. Lett. 2020, 124, 086801. 32167324 10.1103/PhysRevLett.124.086801

[46] L. Li , C. H. Lee , S. Mu , J. Gong , Nat. Commun. 2020, 11, 5491. 33127908 10.1038/s41467-020-18917-4 PMC7603343

[47] C. H. Lee , S. Longhi , Commun. Phys. 2020, 3, 147.

[48] Y. Fu , J. Hu , S. Wan , Phys. Rev. B 2021, 103, 045420.

[49] L. Li , S. Mu , C. H. Lee , J. Gong , Nat. Commun. 2021, 12, 5294. 34489421 10.1038/s41467-021-25626-z PMC8421445

[50] T. Helbig , T. Hofmann , S. Imhof , M. Abdelghany , T. Kiessling , L. W. Molenkamp , C. H. Lee , A. Szameit , M. Greiter , R. Thomale , Nat. Phys. 2020, 16, 747.

[51] T. Hofmann , T. Helbig , F. Schindler , N. Salgo , M. Brzezińska , M. Greiter , T. Kiessling , D. Wolf , A. Vollhardt , A. Kabaši , C. H. Lee , A. Bilušić , R. Thomale , T. Neupert , Phys. Rev. Res. 2020, 2, 023265.

[52] S. Liu , R. Shao , S. Ma , L. Zhang , O. You , H. Wu , Y. J. Xiang , T. J. Cui , S. Zhang , Research 2021, 2021, 5608038. 33824952 10.34133/2021/5608038 PMC7989004

[53] X. Zhang , Y. Tian , J.‐H. Jiang , M.‐H. Lu , Y.‐F. Chen , Nat. Commun. 2021, 12, 5377. 34508089 10.1038/s41467-021-25716-y PMC8433224

[54] L. S. Palacios , S. Tchoumakov , M. Guix , I. Pagonabarraga , S. Sánchez , A. G. Grushin , Nat. Commun. 2021, 12, 4691. 34344869 10.1038/s41467-021-24948-2 PMC8333048

[55] S. Weidemann , M. Kremer , T. Helbig , T. Hofmann , A. Stegmaier , M. Greiter , R. Thomale , A. Szameit , Science 2020, 368, 311. 32217752 10.1126/science.aaz8727

[56] G. Carleo , I. Cirac , K. Cranmer , L. Daudet , M. Schuld , N. Tishby , L. Vogt‐Maranto , L. Zdeborová , Rev. Mod. Phys. 2019, 91, 045002.

[57] P. Mehta , M. Bukov , C.‐H. Wang , A. G. Day , C. Richardson , C. K. Fisher , D. J. Schwab , Phys. Rep. 2019, 810, 1. 31404441 10.1016/j.physrep.2019.03.001 PMC6688775

[58] M. Buchanan , Nature 2019, 15, 1208.

[59] G. E. Karniadakis , I. G. Kevrekidis , L. Lu , P. Perdikaris , S. Wang , L. Yang , Nat. Rev. Phys. 2021, 3, 422.

[60] W. Kritzinger , M. Karner , G. Traar , J. Henjes , W. Sihn , IFAC‐PapersOnLine 2018, 51, 1016.

[61] F. Tao , Q. Qi , Nature 2019, 573, 490. 31554984 10.1038/d41586-019-02849-1

[62] M. Singh , E. Fuenmayor , E. P. Hinchy , Y. Qiao , N. Murray , D. Devine , Appl. Syst. Innov. 2021, 4, 36.

[63] D. H. Gelernter , Mirror Worlds: or, the Day Software Puts the Universe in a Shoebox: How It Will Happen and What It Will Mean, Oxford University Press, Oxford 1993.

[64] M. W. Grieves , Int. J. Prod. Dev. 2005, 2, 71.

[65] D. M. Pozar , Microwave Engineering, John Wiley & Sons, Hoboken, NJ 2011.

[66] D. B. West , Introduction to Graph Theory, Prentice Hall, Hoboken, NJ 2001.

[67] A. Likas , N. Vlassis , J. J. Verbeek , Pattern Recognit. 2003, 36, 451.

[68] J. Wu , Advances in K‐Means Clustering: A Data Mining Thinking, Springer, Berlin 2012.

[69] G. H. Moore , Zermelo's Axiom of Choice: Its Origins, Development, and Influence, Springer, Berlin 1982.

[70] G. L. Miller , J. Comput. Syst. Sci. 1979, 18, 128.

[71] K. B. Petersen , Pedersen, The Matrix Cookbook, Technical University of Denmark, Lyngby 2008.

[72] Z. Gong , Y. Ashida , K. Kawabata , K. Takasan , S. Higashikawa , M. Ueda , Phys. Rev. X 2018, 8, 031079.

[73] S. Hayashi , Commun. Math. Phys. 2018, 364, 343.

[74] S. Hayashi , Lett. Math. Phys. 2019, 109, 2223.

[75] R. Okugawa , S. Hayashi , T. Nakanishi , Phys. Rev. B 2019, 100, 235302.

[76] J. Ningyuan , C. Owens , A. Sommer , D. Schuster , J. Simon , Phys. Rev. X 2015, 5, 021031.

[77] C. H. Lee , A. Sutrisno , T. Hofmann , T. Helbig , Y. Liu , Y. S. Ang , L. K. Ang , X. Zhang , M. Greiter , R. Thomale , Nat. Commun. 2020, 11, 4385. 32873794 10.1038/s41467-020-17716-1 PMC7463261

