# Supplementary material for: Experimental Identification of the Second‐Order Non‐Hermitian Skin Effect with Physics‐Graph‐Informed Machine Learning
Source: Adv Sci (Weinh). 2022 Nov 13;9(36):2202922. doi: 10.1002/advs.202202922 (PMC9799024; doi:10.1002/advs.202202922)
Supplement: Supplementary file 1 — Supporting Information [file ADVS-9-2202922-s001.pdf]

ADVANCED SCIENCE
Open Access

Supporting Information

for *Adv. Sci.*, DOI 10.1002/advs.202202922

Experimental Identification of the Second-Order Non-Hermitian Skin Effect with Physics-Graph-Informed Machine Learning

Ce Shang, Shuo Liu, Ruiwen Shao, Peng Han, Xiaoning Zang, Xiangliang Zhang, Khaled Nabil Salama, Wenlong Gao, Ching Hua Lee, Ronny Thomale, Aurélien Manchon, Shuang Zhang*, Tie Jun Cui* and Udo Schwingenschlögl**

Supplementary Material for

Experimental identification of the second-order non-Hermitian skin effect with physics-graph-informed machine learning

Ce Shang,[1,*] Shuo Liu,[2,*] Ruiwen Shao,[2,*] Peng Han,[3] Xiaoning Zang,[1] Xiangliang Zhang,[4,3] Khaled Nabil Salama,[3] Wenlong Gao,[5] Ching Hua Lee,[6] Ronny Thomale,[7] Aurélien Manchon,[8,†] Shuang Zhang,[9,‡] Tie Jun Cui,[2,§] and Udo Schwingenschlögl[1,¶]

[1]*King Abdullah University of Science and Technology (KAUST), Physical Science and Engineering Division (PSE), Thuwal 23955-6900, Saudi Arabia.*

[2]*State Key Laboratory of Millimeter Waves, Southeast University, Nanjing 210096, China.*

[3]*King Abdullah University of Science and Technology (KAUST), Computer, Electrical, and Mathematical Sciences and Engineering Division (CEMSE), Thuwal 23955-6900, Saudi Arabia.*

[4]*Department of Computer Science and Engineering, University of Notre Dame, Notre Dame, IN 46556, USA*

[5]*Paderborn University, Department of Physics, Warburger Str. 100, 33098 Paderborn, Germany*

[6]*Department of Physics, National University of Singapore, Singapore 117551, Republic of Singapore*

[7]*Institut für Theoretische Physik und Astrophysik, Universitt Wrzburg, Würzburg, Germany.*

[8]*CINaM, Aix-Marseille University, CNRS, Marseille, France.*

[9]*Department of Physics, The University of Hong Kong, Hong Kong, China*

This document includes:

Supplementary Sections S1 to S5

Supplementary Figures S1 to S9

Supplementary Tables S1 and S2

Supplementary Algorithms S1 and S2

* These authors contributed equally

† manchon@cinam.univ-mrs.fr

‡ shuzhang@hku.hk

§ tjcui@seu.edu.cn

¶ udo.schwingenschlogl@kaust.edu.sa

CONTENTS

S1. Non-Hermitian circuit 3

A. Circuit Laplacian formulation 3

B. Impedance, admittance, and scattering matrices 4

C. Non-Hermitian circuit 6

S2. Boundary modes 7

A. Generalized Bloch band theory 7

B. OBC in the y-direction 10

C. OBC in the x-direction 11

D. Skin corner modes 13

E. Spectra of the edge modes 15

S3. Physics-graph-informed machine learning 16

A. From graph to circuit 16

B. K-means method 17

C. Reconstruction 18

D. Execution of the PGIML method 18

S4. Experimental implementation 22

A. Experimental setup 22

B. Fitting model 23

C. Selection of measurements 23

References 30

S1. NON-HERMITIAN CIRCUIT

To realize the non-Hermitian skin effect (NHSE) in topoelectrical circuits, we start with the existing Hermitian formulation for grounded circuit Laplacians [1] and obtain a general description for non-Hermitian circuits.

A. Circuit Laplacian formulation

According to Kirchhoffs laws, the response of an electrical circuit can be described by the equation of motion

$$\frac{d}{dt}I_a = C_{ab}\frac{d^2}{dt^2}V_b + W_{ab}V_b, \tag{S1}$$

where I_a is the current flowing out of node a and V_b is the electrical potential at node b. C_{ab} and W_{ab} are the capacitance and conductance between nodes a and b, respectively. When we apply an alternating voltage $V(t) = V(0)e^{\mathrm{i}\omega t}$ to the circuit, Eq. (S1) yields

$$I_a = \sum_b (\mathrm{i}\omega C_{ab} + \frac{1}{\mathrm{i}\omega}W_{ab})V_b = \sum_b J_{ab}(\omega)V_b. \tag{S2}$$

In matrix form, we have

$$\boldsymbol{J}(\omega) = \mathrm{i}\omega\boldsymbol{C} + \frac{1}{\mathrm{i}\omega}\boldsymbol{W}, \tag{S3}$$

where $\boldsymbol{J}(\omega)$ is the circuit Laplacian and ω is the frequency. $\boldsymbol{C}$ and $\boldsymbol{W}$ are the Laplacian matrices of the capacitance and inverse inductance, respectively. The diagonal and off-diagonal elements represent the self-admittance via a certain node and mutual admittance between two nodes, respectively. We use ω for steady-state analysis of the circuit and obtain an adiabatic continuum of spectra $j(\omega)$ corresponding to $\boldsymbol{J}(\omega)$. As the capacitance and inductance explicitly depend on it, the frequency ω of the driving voltage is a central tuning parameter of topolectrical circuits.

Due to the translational symmetry and Bloch's theorem, a circuit can be represented by periodically repeated cells labelled by the coordinate vector $\boldsymbol{r}$ with the additional sublattice degree of freedom $\alpha \in 1, 2, \ldots, M$. Discrete translational invariance leads to a Laplacian $J_{(\boldsymbol{r},\alpha),(\boldsymbol{r}',\alpha')} = J_{\alpha,\alpha'}(\boldsymbol{r}-\boldsymbol{r}')$ that only depends on the distance between the cells. Thus, the Bloch Hamiltonian of the unit cell is diagonalized by the spatial Fourier transformation

$$\boldsymbol{J}(\boldsymbol{k},\omega) = \sum_{\boldsymbol{r}} e^{-\mathrm{i}\boldsymbol{k}\cdot\boldsymbol{r}}\boldsymbol{J}(\boldsymbol{r},\omega), \tag{S4}$$

resulting in

$$\boldsymbol{I}(\boldsymbol{k},\omega) = \boldsymbol{J}(\boldsymbol{k},\omega)\,\boldsymbol{V}(\boldsymbol{k},\omega). \tag{S5}$$

By diagonalizing the $N \times N$ matrix $\boldsymbol{J}(\boldsymbol{k},\omega)$, we obtain N eigenvalues, which constitute the band structure $j(\boldsymbol{k},\omega)$ of the complex-valued admittance, parametrically depending on $\boldsymbol{k}$ and ω.

B. Impedance, admittance, and scattering matrices

A circuit is most commonly studied through measurement of the two-point impedance [2]

$$Z_{ab} = \frac{V_a - V_b}{I_{ab}}, \tag{S6}$$

where I_{ab} is the current between nodes a and b. We can use the impedance or admittance matrices to relate the nodes to each other and arrive at a matrix description of the circuit [3, 4]. We begin by considering an arbitrary N-node circuit with the admittance matrix $\boldsymbol{J}$ defined by

$$\begin{bmatrix} I_1 \\ I_2 \\ \vdots \\ I_N \end{bmatrix} = \begin{bmatrix} J_{11} & J_{12} & \dots & J_{1N} \\ J_{21} & J_{22} & \dots & J_{2N} \\ \vdots & \vdots & \ddots & \vdots \\ J_{N1} & J_{N2} & \dots & J_{NN} \end{bmatrix} \begin{bmatrix} V_1 \\ V_2 \\ \vdots \\ V_N \end{bmatrix}, \tag{S7}$$

or in matrix form

$$\boldsymbol{I} = \boldsymbol{J}\boldsymbol{V}. \tag{S8}$$

The impedance matrix $\boldsymbol{Z}$ is defined by

$$\begin{bmatrix} V_1 \\ V_2 \\ \vdots \\ V_N \end{bmatrix} = \begin{bmatrix} Z_{11} & Z_{12} & \dots & Z_{1N} \\ Z_{21} & Z_{22} & \dots & Z_{2N} \\ \vdots & \vdots & \ddots & \vdots \\ Z_{N1} & Z_{N2} & \dots & Z_{NN} \end{bmatrix} \begin{bmatrix} I_1 \\ I_2 \\ \vdots \\ I_N \end{bmatrix}, \tag{S9}$$

or in matrix form

$$\boldsymbol{V} = \boldsymbol{Z}\boldsymbol{I}. \tag{S10}$$

At the a-th node, the voltages (V_a^+, V_a^-) and currents (I_a^+, I_a^-) of the incident $(+)$ and reflected $(-)$ waves are given by

$$V_a = V_a^+ + V_a^-, \tag{S11a}$$

$$I_a = I_a^+ - I_a^- = \frac{V_a^+ - V_a^-}{Z_{0a}}, \tag{S11b}$$

where Z_{0a} is the characteristic impedance of node a.

The scattering matrix $\boldsymbol{S}$ (S-matrix) is defined by

$$\begin{bmatrix} V_1^- \\ V_2^- \\ \vdots \\ V_N^- \end{bmatrix} = \begin{bmatrix} S_{11} & S_{12} & \dots & S_{1N} \\ S_{21} & S_{22} & \dots & S_{2N} \\ \vdots & \vdots & \ddots & \vdots \\ S_{N1} & S_{N2} & \dots & S_{NN} \end{bmatrix} \begin{bmatrix} V_1^+ \\ V_2^+ \\ \vdots \\ V_N^+ \end{bmatrix}, \tag{S12}$$

or in matrix form

$$\boldsymbol{V}^- = \boldsymbol{S}\boldsymbol{V}^+. \tag{S13}$$

The elements of the S-matrix (S-parameters),

$$S_{ab} = \left.\frac{V_a^-}{V_b^+}\right|_{V_c^+=0 \text{ for } c\neq b}, \tag{S14}$$

are found by applying an incident wave of voltage V_b^+ to node b and measuring the voltage of the reflected wave V_a^- at node a. The incident waves are set to zero at all the other nodes, which thus must be terminated with matched loads to avoid reflections. Consequently, S_{aa} is the reflection coefficient of node a and S_{ab} is the transmission coefficient from node a to node b.

We assume that the characteristic impedances of all the nodes are identical,

$$Z_{01} = Z_{02} = \cdots = Z_{0N} = Z_0. \tag{S15}$$

Equations (S10), (S11a), and (S11b) imply

$$\boldsymbol{V} = \boldsymbol{V}^+ + \boldsymbol{V}^- = \boldsymbol{Z}\boldsymbol{I} = \boldsymbol{Z}(\boldsymbol{I}^+ - \boldsymbol{I}^-) = \boldsymbol{Z}\frac{\boldsymbol{V}^+ - \boldsymbol{V}^-}{Z_0}, \tag{S16}$$

which can be rewritten as

$$(\mathbb{I} + \frac{1}{Z_0}\boldsymbol{Z})\,\boldsymbol{V}^- = (\frac{1}{Z_0}\boldsymbol{Z} - \mathbb{I})\,\boldsymbol{V}^+, \tag{S17}$$

where $\mathbb{I}$ is the identity matrix. Using Eq. (S13), we obtain

$$\boldsymbol{S} = (\boldsymbol{Z} + Z_0\mathbb{I})^{-1}(\boldsymbol{Z} - Z_0\mathbb{I}) \tag{S18}$$

and

$$\boldsymbol{Z} = Z_0(\boldsymbol{S} + \mathbb{I})(\mathbb{I} - \boldsymbol{S})^{-1}. \tag{S19}$$

The impedance matrix, admittance matrix, and S-matrix each provide a complete description of the circuit. While the impedance and admittance matrices relate the total voltages to the total currents at the nodes, the S-matrix relates the voltages of the waves incident to the nodes to those of the waves reflected from the nodes. The S-parameters can be measured directly by a vector network analyzer. Once they are known, conversion to other matrix parameters can be performed.

C. Non-Hermitian circuit

We design a circuit without Hermiticity and reciprocity to study the second-order NHSE. The unit cell with nodes A and B is schematically depicted in Fig. 2d (main text) and the corresponding circuit board is shown in Fig. 2b (main text). The circuit contains intracell connections of capacitance C_2 in the y-direction, intercell connections of capacitance C_1 in the y-direction, intercell non-reciprocal connections of capacitance C_1 connected to a voltage follower in the x-direction, and intercell non-reciprocal connections of inductance L_1 reversely connected to a voltage follower in the x-direction. The currents out of the nodes relate to the voltages at the nodes as

$$\begin{aligned}
I_\mathrm{A}(\boldsymbol{r}) &= \mathrm{i}\omega \left\{ C_1 \left[V_\mathrm{B}(\boldsymbol{r}) - V_\mathrm{A}(\boldsymbol{r})\right] + C_2 \left[V_\mathrm{B}(\boldsymbol{r}+\boldsymbol{y}) - V_\mathrm{A}(\boldsymbol{r})\right] + C_1 \left[V_\mathrm{A}(\boldsymbol{r}+\boldsymbol{x}) - V_\mathrm{A}(\boldsymbol{r})\right] - \frac{V_\mathrm{A}(\boldsymbol{r})}{\omega^2 G_\mathrm{A}} \right\}, \\
I_\mathrm{B}(\boldsymbol{r}) &= \mathrm{i}\omega \left\{ C_1 \left[V_\mathrm{A}(\boldsymbol{r}) - V_\mathrm{B}(\boldsymbol{r})\right] + C_2 \left[V_\mathrm{A}(\boldsymbol{r}-\boldsymbol{y}) - V_\mathrm{B}(\boldsymbol{r})\right] - \frac{V_\mathrm{B}(\boldsymbol{r}-\boldsymbol{x}) - V_\mathrm{B}(\boldsymbol{r})}{\omega^2 L_1} - \frac{V_\mathrm{B}(\boldsymbol{r})}{\omega^2 G_\mathrm{B}} \right\},
\end{aligned} \tag{S20}$$

with the position vector $\boldsymbol{r} = (m, n)$, lattice vectors $\boldsymbol{x} = (1, 0)$ and $\boldsymbol{y} = (0, 1)$, and grounding components $G_\mathrm{A} = 2L_1L_2/(2L_1 + L_2)$ and $G_\mathrm{B} = L_2$. We obtain

$$\begin{pmatrix} I_\mathrm{A} \\ I_\mathrm{B} \end{pmatrix} = \boldsymbol{J}(\boldsymbol{r}, \omega) \begin{pmatrix} V_\mathrm{A} \\ V_\mathrm{B} \end{pmatrix} \tag{S21}$$

with

$$\boldsymbol{J}(\boldsymbol{r}, \omega) = \mathrm{i}\omega \begin{bmatrix} \left(-2C_1 - C_2 + \frac{2}{\omega^2 L_1} + \frac{1}{\omega^2 L_2}\right) \delta_{\boldsymbol{r},(0,0)} + C_1 \delta_{\boldsymbol{r},(1,0)} & C_2 \delta_{\boldsymbol{r},(0,0)} + C_1 \delta_{\boldsymbol{r},(0,1)} \\ C_2 \delta_{\boldsymbol{r},(0,0)} + C_1 \delta_{\boldsymbol{r},(0,-1)} & \left(-C_1 - C_2 + \frac{1}{\omega^2 L_1} + \frac{1}{\omega^2 L_2}\right) \delta_{\boldsymbol{r},(0,0)} - \frac{\delta_{\boldsymbol{r},(-1,0)}}{\omega^2 L_1} \end{bmatrix}, \tag{S22}$$

where $\delta_{\boldsymbol{r},\boldsymbol{r}'}$ is the two-dimensional (2D) discrete delta function. We arrive at the non-reciprocal two-band admittance matrix

$$\boldsymbol{J}(\boldsymbol{k},\omega) = \mathrm{i}\omega \begin{bmatrix} \frac{L_1L_2}{(L_1+2L_2)\omega^2} - 2C_1 - C_2 + C_1e^{-\mathrm{i}k_x} & C_2 + C_1e^{-\mathrm{i}k_y} \\ C_2 + C_1e^{\mathrm{i}k_y} & \frac{L_1L_2}{(L_1+L_2)\omega^2} - C_1 - C_2 - \frac{L_1}{\omega^2}e^{\mathrm{i}k_x} \end{bmatrix}, \tag{S23}$$

where two pairs of capacitors and inductors, (C_1, L_1) and (C_2, L_2), with the same resonance frequency $\omega_0 = 1/\sqrt{L_1C_1} = 1/\sqrt{L_2C_2}$ are used to couple the nodes. Equation (S23) implies

$$\begin{aligned}\boldsymbol{J}(\boldsymbol{k},\omega_0) &= \mathrm{i}\sqrt{C_1/L_1}\left[-\mathrm{i}\lambda_x \sin k_x\sigma_0 + \lambda_x\cos k_x\sigma_z + \lambda_y \sin k_y\sigma_y + (\gamma_y + \lambda_y\cos k_y)\sigma_x\right] \\ &=: \mathrm{i}\sqrt{C_1/L_1}H(\mathbf{k}).\end{aligned} \tag{S24}$$

For $C_1 = 1000\,\mathrm{pH}$, $C_2 = 330\,\mathrm{pH}$, $L_1 = 33\,\mu\mathrm{F}$, and $L_2 = 100\,\mu\mathrm{F}$, we arrive at $\lambda_x = 1$, $\lambda_y = 1$, and $\gamma_y = 0.33$.

The circuit Laplacian can be represented by the real-space tight-binding Hamiltonian

$$H = \lambda_x \sum_{\boldsymbol{r}} (c^\dagger_{\boldsymbol{r}+\boldsymbol{x},\mathrm{A}}c_{\boldsymbol{r},\mathrm{A}} - c^\dagger_{\boldsymbol{r},\mathrm{B}}c_{\boldsymbol{r}-\boldsymbol{x},\mathrm{B}}) + \sum_{\boldsymbol{r}} (\lambda_y c^\dagger_{\boldsymbol{r},\mathrm{A}}c_{\boldsymbol{r}+\boldsymbol{y},\mathrm{B}} + \gamma_y c^\dagger_{\boldsymbol{r},\mathrm{A}}c_{\boldsymbol{r},\mathrm{B}} + h.c.), \tag{S25}$$

where $c^\dagger_{\boldsymbol{x},\alpha}$ $(c_{\boldsymbol{x},\alpha})$ is the creation (annihilation) operator of sublattice α in the unit cell at position $\boldsymbol{r}$, λ_x and λ_y are the intracell hopping amplitudes, and γ_y is the intercell hopping amplitude.

S2. BOUNDARY MODES

We solve the non-Hermitian Hamiltonian of Eq. (S25) for different boundary conditions and obtain the edge and corner skin modes.

A. Generalized Bloch band theory

The bulk properties of an open boundary condition (OBC) Hamiltonian can be characterized by a Bloch periodic boundary condition (PBC) Hamiltonian [5]. As the OBC Hamiltonian may host the NHSE [6, 7], the spectra of the OBC and PBC Hamiltonians can be different. In both Hermitian and non-Hermitian systems, the OBC Hamiltonian can be encoded using the generalized Brillouin zone [8–10] (although the OBC breaks the translational symmetry) by providing a generalized Bloch Hamiltonian for which the boundary scattering can be regarded as a perturbation. We use the generalized Bloch band theory to determine the generalized Brillouin zone C_β for $\beta := e^{\mathrm{i}k}, k \in \mathbb{C}$.

For a one-dimensional (1D) tight-binding OBC Hamiltonian with a unit cell of q degrees of freedom and hopping of any range, we can write

$$H = \sum_n \sum_{i=-N}^{N} \sum_{\mu,\nu=1}^{q} t_{i,\mu\nu} c^\dagger_{n+i,\mu} c_{n,\nu}, \tag{S26}$$

where $c^\dagger_{n,\mu}$ ($c_{n,\mu}$) is the creation (annihilation) operator in the n-th unit cell and $t_{i,\mu\nu}$ is the hopping to the i-th nearest unit cell. The eigenvector $|\psi\rangle = (\psi_{1,1}, \psi_{1,2}, \ldots, \psi_{n,q})^T$ resulting from the real-space eigenvalue equation $H|\psi\rangle = E|\psi\rangle$ can be written as a linear combination of wave functions,

$$\psi_{n,\mu} = \sum_j \phi^{(j)}_{n,\mu}. \tag{S27}$$

Since $\phi^{(j)}_{n,\mu} = \beta_j^n \phi^{(j)}_\mu$, we have

$$\psi_{n,\mu} = \beta_j^n \phi^{(j)}_\mu. \tag{S28}$$

$H|\psi\rangle = E|\psi\rangle$ can be rewritten as

$$\sum_{\nu=1}^{q} [\mathcal{H}(\beta)]_{\mu\nu} \phi_\nu = E\phi_\mu \tag{S29}$$

with the generalized Bloch Hamiltonian

$$[\mathcal{H}(\beta)]_{\mu\nu} = \sum_{i=-N}^{N} t_{i,\mu\nu} \beta^i. \tag{S30}$$

The eigenvalue equation is solved as

$$\det\left[\mathcal{H}(\beta) - E\right] = 0, \tag{S31}$$

yielding $2M$ $(= 2qN)$ solutions β_j with

$$|\beta_1| \leq |\beta_2| \leq \cdots \leq |\beta_{2M-1}| \leq |\beta_{2M}|. \tag{S32}$$

For a chain with L unit cells, Eq. (S28) implies

$$\psi_{n,\mu} = \sum_{j=1}^{2M} \beta_j^n \phi^{(j)}_\mu \quad (n = 1, 2, \ldots, L; \mu = 1, 2, \ldots, q). \tag{S33}$$

As the ratio $\phi^{(j)}_\mu \big/ \phi^{(j')}_\mu$ is equal to a constant for all $j \neq j'$, $\mu = 1, 2, \ldots, q$ reduces to a single value $\mu = 1$. As a consequence, we obtain

$$\sum_{j=1}^{2M} f_i(\beta_j, E, \mathcal{D}) \phi^{(j)}_1 = 0 \quad (i = 1, 2, \ldots, M) \tag{S34}$$

at the left end of the chain ($n = 1$) and

$$\sum_{j=1}^{2M} g_i(\beta_j, E, \mathcal{D}) \beta_j^L \phi_1^{(j)} = 0 \quad (i = 1, 2, \ldots, M) \tag{S35}$$

at the right end of the chain ($n = L$), where $\mathcal{D}$ is the set of hopping parameters $t_{i,\mu\nu}$. By combining Eqs. (S34) and (S35), we have to fulfill

$$\det \begin{bmatrix} f_1(\beta_1, E, \mathcal{D}) & \ldots & f_1(\beta_{2M}, E, \mathcal{D}) \\ \vdots & \vdots & \vdots \\ f_M(\beta_1, E, \mathcal{D}) & \ldots & f_M(\beta_{2M}, E, \mathcal{D}) \\ g_1(\beta_1, E, \mathcal{D})\beta_1^L & \ldots & g_1(\beta_{2M}, E, \mathcal{D})\beta_{2M}^L \\ \vdots & \vdots & \vdots \\ g_M(\beta_1, E, \mathcal{D})\beta_1^L & \ldots & g_M(\beta_{2M}, E, \mathcal{D})\beta_{2M}^L \end{bmatrix} = 0 \tag{S36}$$

for a nontrivial solution. Equation (S36) is an algebraic equation for β_j and, for L sufficiently large, can be written as

$$\sum_{P,Q} F(\beta_{i\in P}, \beta_{j\in Q}, E, \mathcal{D}) \prod_{k\in P} \beta_k^L = 0, \tag{S37}$$

where P and Q are two disjoint subsets of $\{1, 2, \ldots, 2M\}$. As L grows, the energy levels become dense and asymptotically continuous. The asymptotic distribution of β is the generalized Brillouin zone C_β. In Hermitian systems, β equals unity, implying that the eigenmodes extend over the bulk. In non-Hermitian systems, β does not necessarily equal unity and the eigenmodes may be localized at either end of the chain. To distinguish them from the bulk bands, they are called the continuum bands.

We next consider the asymptotic behavior of the solutions of Eq. (S37) for large L. When $|\beta_M| \neq |\beta_{M+1}|$, we have a leading term of Eq. (S37) proportional to $(\beta_M \beta_{M+1} \ldots \beta_{2M})^L$, and obtain

$$F(\beta_{i\in P_0}, \beta_{j\in Q_0}, E, \mathcal{D}) = 0 \tag{S38}$$

with $P_0 = \{M+1, M+2, \ldots, 2M\}$ and $Q_0 = \{1, 2, \ldots, M\}$. Equation (S38) does not depend on L and does not include the continuum bands. When $|\beta_M| = |\beta_{M+1}|$, we have leading terms of Eq. (S37) proportional to $(\beta_M \beta_{M+2} \ldots \beta_{2M})^L$ and $(\beta_{M+1} \beta_{M+2} \ldots \beta_{2M})^L$, and obtain

$$\frac{F(\beta_{i\in P_1}, \beta_{j\in Q_1}, E, \mathcal{D})}{F(\beta_{i\in P_2}, \beta_{j\in Q_2}, E, \mathcal{D})} = -\left(\frac{\beta_M}{\beta_{M+1}}\right)^L \tag{S39}$$

with $P_1 = \{M+1, M+2, \ldots, 2M\}$, $Q_1 = \{1, 2, \ldots, M\}$, $P_2 = \{M, M+2, \ldots, 2M\}$, and $Q_2 = \{1, 2, \ldots, M-1, M+1\}$. The phase between β_M and β_{M+1} changes almost continuously for large L, giving rise to the continuum bands. The trajectories of β_M and β_{M+1} with $|\beta_M| = |\beta_{M+1}|$ determine C_β. Irrespective of the boundary condition, the spectrum of a long chain asymptotically approaches the continuum bands of C_β. When the numbering of the sites is reversed by setting $n' = L + 1 - n$ for $n = 1, 2, \ldots, L$ and replacing β by $\beta' = 1/\beta$, then $|\beta'_M| = \left|\beta'_{M+1}\right|$.

The described method can be extended to higher dimensions. In 2D systems, for example, two parameters $\beta_x := e^{\mathrm{i}k_x}$ with $k_x \in \mathbb{C}$ and $\beta_y := e^{\mathrm{i}k_y}$ with $k_y \in \mathbb{C}$ are introduced. Then the eigenvalue equation is given by $\det\left[\mathcal{H}(\beta_x, \beta_y) - E\right] = 0$, where $\mathcal{H}(\beta_x, \beta_y)$ is a 2D generalized Bloch Hamiltonian. If we fix β_y (β_x), we have a 1D system and $|\beta_{x,M_x}| = |\beta_{x,M_x+1}|$ ($|\beta_{y,M_y}| = |\beta_{y,M_y+1}|$), where $2M_x$ ($2M_y$) is the dimension of the eigenvalue equation for β_x (β_y).

B. OBC in the y-direction

We consider a semi-infinite 2D lattice that has edges in the y-direction. Equation (S25) can be written in terms of the momentum k_x in the x-direction and the real space lattice index n in the y-direction (k_y is no longer a good quantum number, since the system is not translational invariant in the y-direction). Fourier transformation with respect to k_y yields a 1D tight-binding Hamiltonian that depends only on k_x [11],

$$H_{k_x} = \sum_{k_x,n} (\Psi^\dagger_{k_x,n} M_{k_x} \Psi_{k_x,n} + \Psi^\dagger_{k_x,n+1} T_{y-} \Psi_{k_x,n} + \Psi^\dagger_{k_x,n} T_{y+} \Psi_{k_x,n-1}) \tag{S40}$$

with

$$M_{k_x} = -\mathrm{i}\lambda_x \sin k_x \sigma_0 + \lambda_x \cos k_x \sigma_z + \gamma_y \sigma_x, \tag{S41a}$$

$$T_{y\pm} = \lambda_y \frac{\sigma_x \pm \mathrm{i}\sigma_y}{2}. \tag{S41b}$$

We construct Harpers equation for the 2D wave function as

$$M_{k_x} \Psi_{k_x,n} + T_{y-} \Psi_{k_x,n+1} + T_{y+} \Psi_{k_x,n-1} = E_{k_x} \Psi_{k_x,n} \tag{S42}$$

with the boundary conditions

$$M_{k_x} \Psi_{k_x,1} + T_{y-} \Psi_{k_x,2} = E_{k_x} \Psi_{k_x,1}, \tag{S43a}$$

$$M_{k_x} \Psi_{k_x,L_y} + T_{y+} \Psi_{k_x,L_y-1} = E_{k_x} \Psi_{k_x,L_y}. \tag{S43b}$$

Equations (S43a) and (S43b) can be simplified as

$$T_{y+}\Psi_{k_x,0} = T_{y-}\Psi_{k_x,L_y+1} = 0. \tag{S44}$$

The generalized Bloch Hamiltonian is

$$\mathcal{H}_{k_x}(\beta_y) = M_{k_x} + \beta_y T_{y-} + \beta_y^{-1} T_{y+} \tag{S45}$$

and we obtain the eigenvalue equation

$$E_{k_x}^2 + 2\mathrm{i}\lambda_x E_{k_x}\sin k_x - (\beta_y + \beta_y^{-1})\lambda_y\gamma_y - \lambda_x^2 - \lambda_y^2 - \gamma_y^2 = 0, \tag{S46}$$

which results in two eigenvalues $\beta_{y\pm}$ for given k_x and E_{k_x}. The corresponding eigenvectors are

$$\Phi_{y\pm} = \begin{pmatrix} \dfrac{E_{k_x} + \lambda_x e^{\mathrm{i}k_x}}{\sqrt{(E_{k_x} + \lambda_x e^{\mathrm{i}k_x})^2 + (\gamma_y + \beta_{y\pm}\lambda_y)^2}} \\ \dfrac{\gamma_y + \beta_{y\pm}\lambda_y}{\sqrt{(E_{k_x} + \lambda_x e^{\mathrm{i}k_x})^2 + (\gamma_y + \beta_{y\pm}\lambda_y)^2}} \end{pmatrix}. \tag{S47}$$

To fulfill Eq. (S44), Φ_{y+} and Φ_{y-} must be linearly dependent. Therefore, we can reduce the condition for the existence of edge modes as

$$\det\left[\Phi_{y+} \quad \Phi_{y-}\right] = 0. \tag{S48}$$

Combining Eqs. (S46) and (S48) implies

$$E_{k_x} = -\lambda_x e^{\mathrm{i}k_x}, \tag{S49a}$$

$$\beta_y = -\frac{\gamma_y}{\lambda_y}. \tag{S49b}$$

The edge modes are localized for $|\beta_y| < 1$ and no edge modes exist for $|\beta_y| \geq 1$.

C. OBC in the x-direction

Following the procedure of Sec. S2 B, Fourier transformation with respect to k_x yields a 1D tight-binding Hamiltonian that depends only on k_y,

$$H_{k_y} = \sum_{k_y,m} (\Psi_{k_y,m}^\dagger M_{k_y}\Psi_{k_y,m} + \Psi_{k_y,m+1}^\dagger T_{x-}\Psi_{k_y,m} + \Psi_{k_y,m}^\dagger T_{x+}\Psi_{k_y,m-1}) \tag{S50}$$

with

$$M_{k_y} = (\gamma_y + \lambda_y\cos k_y)\sigma_x + \lambda_y\sin k_y\sigma_y, \tag{S51a}$$

$$T_{x\pm} = \lambda_x\frac{\sigma_0 \pm \sigma_z}{2}. \tag{S51b}$$

We construct Harpers equation for the 2D wave function as

$$M_{k_y}\Psi_{k_y,m} + T_{x-}\Psi_{k_y,m+1} + T_{x+}\Psi_{k_y,m-1} = E_{k_y}\Psi_{k_y,m} \tag{S52}$$

with the boundary conditions

$$M_{k_y}\Psi_{k_y,1} + T_{x-}\Psi_{k_x,2} = E_{k_y}\Psi_{k_y,1}, \tag{S53a}$$

$$M_{k_y}\Psi_{k_y,L_x} + T_{x+}\Psi_{k_y,L_x-1} = E_{k_y}\Psi_{k_y,L_x}. \tag{S53b}$$

Equations (S53a) and (S53b) can be simplified as

$$T_{x+}\Psi_{k_y,0} = T_{x-}\Psi_{k_y,L_x+1} = 0. \tag{S54}$$

The generalized Bloch Hamiltonian is

$$\mathcal{H}_{k_y}(\beta_x) = M_{k_y} + \beta_x T_{x-} + \beta_x^{-1} T_{x+} \tag{S55}$$

and we obtain the eigenvalue equation

$$E_{k_y}^2 + (\beta_x - \beta_x^{-1})\lambda_x E_{k_y} - 2\lambda_y\gamma_y\cos k_y - \lambda_x^2 - \lambda_y^2 - \gamma_y^2 = 0, \tag{S56}$$

which results in two eigenvalues $\beta_{x\pm}$ for given k_y and E_{k_y}. The corresponding eigenvectors are

$$\Phi_{x\pm} = \begin{pmatrix} \dfrac{E_{k_y} + \lambda_x\beta_{x\pm}}{\sqrt{(E_{k_y} + \lambda_x\beta_{x\pm})^2 + (\gamma_y + e^{ik_y}\lambda_y)^2}} \\ \dfrac{\gamma_y + e^{\mathrm{i}k_y}\lambda_y}{\sqrt{(E_{k_y} + \lambda_x\beta_{x\pm})^2 + (\gamma_y + e^{ik_y}\lambda_y)^2}} \end{pmatrix}. \tag{S57}$$

To fulfill Eq. (S54), Φ_{x+} and Φ_{x-} must be linearly dependent. Therefore, we can reduce the condition for the existence of edge modes as

$$\det\left[\Phi_{x+} \quad \Phi_{x-}\right] = 0. \tag{S58}$$

Combining Eqs. (S56) and (S58) implies

$$e^{\mathrm{i}k_y} = -\frac{\gamma_y}{\lambda_y}, \tag{S59a}$$

$$E_{k_y} = -\lambda_x\beta_x. \tag{S59b}$$

Equation (S59a) implies $e^{\mathrm{i}k_y} \in \mathbb{Z}$, i.e., $k_y = 0$ or π with $E_0 = E_\pi$.

D. Skin corner modes

The real-space Hamiltonian for the skin corner modes can be written as

$$H=\sum_{m,n}(\Psi_{m,n}^{\dagger}M\Psi_{m,n}+\Psi_{m+1,n}^{\dagger}T_{x-}\Psi_{m,n}+\Psi_{m,n}^{\dagger}T_{x+}\Psi_{m-1,n}+\Psi_{m,n+1}^{\dagger}T_{y-}\Psi_{m,n}+\Psi_{m,n}^{\dagger}T_{y+}\Psi_{m,n-1}) \tag{S60}$$

with

$$M=\gamma_y\sigma_x, \tag{S61a}$$
$$T_{x\pm}=\lambda_x\frac{\sigma_0\pm\sigma_z}{2}, \tag{S61b}$$
$$T_{y\pm}=\lambda_y\frac{\sigma_x\pm \mathrm{i}\sigma_y}{2}. \tag{S61c}$$

We construct Harpers equation for the 2D wave function as

$$M\Psi_{m,n}+T_{x-}\Psi_{m+1,n}+T_{x+}\Psi_{m-1,n}+T_{y-}\Psi_{m,n+1}+T_{y+}\Psi_{m,n-1}=E\Psi_{m,n} \tag{S62}$$

with the boundary conditions

$$M\Psi_{1,1}+T_{x-}\Psi_{2,1}+T_{y-}\Psi_{1,2}=E\Psi_{1,1}, \tag{S63a}$$
$$M\Psi_{1,L_y}+T_{x-}\Psi_{2,L_y}+T_{y+}\Psi_{1,L_y-1}=E\Psi_{1,L_y}, \tag{S63b}$$
$$M\Psi_{L_x,1}+T_{x+}\Psi_{L_x-1,1}+T_{y-}\Psi_{L_x,2}=E\Psi_{L_x,1}, \tag{S63c}$$
$$M\Psi_{L_x,L_y}+T_{x+}\Psi_{L_x-1,L_y}+T_{y+}\Psi_{L_x,L_y-1}=E\Psi_{L_x,L_y}. \tag{S63d}$$

Equations (S63a) to (S63d) can be simplified as

$$T_{x+}\Psi_{0,n}=0, \tag{S64a}$$
$$T_{x-}\Psi_{L_x+1,n}=0, \tag{S64b}$$
$$T_{y+}\Psi_{m,0}=0, \tag{S64c}$$
$$T_{y-}\Psi_{m,L_y+1}=0. \tag{S64d}$$

The generalized Bloch Hamiltonian is

$$\mathcal{H}(\beta_x,\beta_y)=M+\beta_xT_{x-}+\beta_x^{-1}T_{x+}+\beta_yT_{y-}+\beta_y^{-1}T_{y+} \tag{S65}$$

and we obtain the eigenvalue equation

$$E^2+(\beta_x-\beta_x^{-1})\lambda_xE-(\beta_y+\beta_y^{-1})\lambda_y\gamma_y-\lambda_x^2-\lambda_y^2-\gamma_y^2=0, \tag{S66}$$

which results in two eigenvalues $\beta_{x\pm,y}$ ($\beta_{x,y\pm}$) for given β_y (β_x) and E. The corresponding eigenvectors are

$$\Phi_{x\pm,y} = \begin{pmatrix} \frac{E+\lambda_x\beta_{x\pm}}{\sqrt{(E+\lambda_x\beta_{x\pm})^2+(\gamma_y+\beta_y\lambda_y)^2}} \\ \frac{\gamma_y+\beta_y\lambda_y}{\sqrt{(E+\lambda_x\beta_{x\pm})^2+(\gamma_y+\beta_y\lambda_y)^2}} \end{pmatrix}, \Phi_{x,y\pm} = \begin{pmatrix} \frac{E+\lambda_x\beta_x}{\sqrt{(E+\lambda_x\beta_x)^2+(\gamma_y+\beta_{y\pm}\lambda_y)^2}} \\ \frac{\gamma_y+\beta_{y\pm}\lambda_y}{\sqrt{(E+\lambda_x\beta_x)^2+(\gamma_y+\beta_{y\pm}\lambda_y)^2}} \end{pmatrix}. \tag{S67}$$

To fulfill Eq. (S64), $\Phi_{x+,y}$ ($\Phi_{x,y+}$) and $\Phi_{x-,y}$ ($\Phi_{x,y-}$) must be linearly dependent. Therefore, we can reduce the condition for the existence of corner skin modes as

$$\det\left[\Phi_{x+,y} \quad \Phi_{x-,y}\right] = 0, \tag{S68a}$$

$$\det\left[\Phi_{x,y+} \quad \Phi_{x,y-}\right] = 0. \tag{S68b}$$

Combining Eqs. (S66), (S68a), and (S68b) implies

$$\beta_y = -\frac{\gamma_y}{\lambda_y}, \tag{S69a}$$

$$E = -\lambda_x\beta_x. \tag{S69b}$$

Equations (S68a) and (S68b) can be rewritten as

$$\det\left[\Phi_{x+,y+} \quad \Phi_{x-,y+}\right] = 0, \tag{S70a}$$

$$\det\left[\Phi_{x+,y-} \quad \Phi_{x-,y-}\right] = 0, \tag{S70b}$$

$$\det\left[\Phi_{x+,y+} \quad \Phi_{x+,y-}\right] = 0, \tag{S70c}$$

$$\det\left[\Phi_{x-,y+} \quad \Phi_{x-,y-}\right] = 0. \tag{S70d}$$

Equations (S64a) to (S64d) imply

$$T_{x+}(\Phi_{x+,y+} + \beta_x^{L_x+1}\Phi_{x-,y+}) = T_{x+}(\Phi_{x+,y-} + \beta_x^{L_x+1}\Phi_{x-,y-}) = 0, \tag{S71a}$$

$$T_{x-}(\beta_x^{L_x+1}\Phi_{x+,y+} + \Phi_{x-,y+}) = T_{x-}(\beta_x^{L_x+1}\Phi_{x+,y-} + \Phi_{x-,y-}) = 0, \tag{S71b}$$

$$T_{y+}(\Phi_{x+,y+} + \beta_y^{L_y+1}\Phi_{x+,y-}) = T_{y+}(\Phi_{x-,y+} + \beta_y^{L_y+1}\Phi_{x-,y-}) = 0, \tag{S71c}$$

$$T_{y-}(\beta_y^{L_y+1}\Phi_{x+,y+} + \Phi_{x+,y-}) = T_{y-}(\beta_y^{L_y+1}\Phi_{x-,y+} + \Phi_{x-,y-}) = 0 \tag{S71d}$$

and we obtain

$$\det\begin{bmatrix} E+\lambda_x\beta_x & \beta_x^{2L_x+2}(E+\lambda_x\beta_x) \\ \gamma_y+\beta_y\lambda_y & \beta_y^{2L_y+2}(\gamma_y+\beta_y\lambda_y) \end{bmatrix} = 0. \tag{S72}$$

For sufficiently large L_x and L_y, we have

$$|\beta_x| = |\beta_y| \tag{S73}$$

and can prove

$$\det\left[\Phi_{x+,y+} \quad \Phi_{x-,y-}\right] = 0 \quad \text{and} \quad \det\left[\Phi_{x+,y-} \quad \Phi_{x-,y+}\right] = 0. \tag{S74}$$

The wave function can be written as

$$\Psi_{m,n} = \beta_x^m \beta_y^n \Phi_{x+,y+} + \beta_x^m \beta_y^{L_y+1-n} \Phi_{x+,y-} + \beta_x^{L_x+1-m} \beta_y^n \Phi_{x-,y+} + \beta_x^{L_x+1-m} \beta_y^{L_y+1-n} \Phi_{x-,y-}. \tag{S75}$$

Since we have $|\beta_x| = |\beta_y| \neq 1$, the eigenmodes are localized at the corners (NHSE). With the definition $\beta_x := |\beta_x| e^{\mathrm{i}\theta}, \theta \in [0, 2\pi]$ and Eq. (S69b), the spectrum of these corner skin modes is given by

$$E = -\gamma_y \frac{\lambda_x}{\lambda_y} e^{\mathrm{i}\theta}, \quad \theta \in [0, 2\pi] \tag{S76}$$

and forms a circle in the complex-energy plane.

E. Spectra of the edge modes

We denote the combination of PBC in the x-direction and OBC in the y-direction, for example, as PBCx-OBCy. Considering a ribbon geometry and Eq. (S23) with the parameters given below Eq. (S24), the simulated spectrum $j_{\text{sim.}}$ of the second-order NHSE is plotted in the $j_{\text{sim.}}$-k_x ($j_{\text{sim.}}$-k_y) space in Fig. S1(a) (S1(b)) for PBCx-OBCy (OBCx-PBCy). The color represents the inverse participation ratio IPR $= \sum_n |\Psi_{n,k}|^4 / (\sum_n |\Psi_{n,k}|^2)^2$, where a larger value corresponds to a more localized wavefunction. For simplicity, all spectra are given in normalized units (nu.), i.e., in multiples of $\sqrt{L_1/C_1}\,\Omega^{-1}$. In contrast to the first-order topological insulator, second-order topological insulator, and first-order NHSE, the second-order NHSE has a gapless edge spectrum in the y-direction, see Fig. S1(a), and has no edge spectrum in the x-direction, see Fig. S1(b).

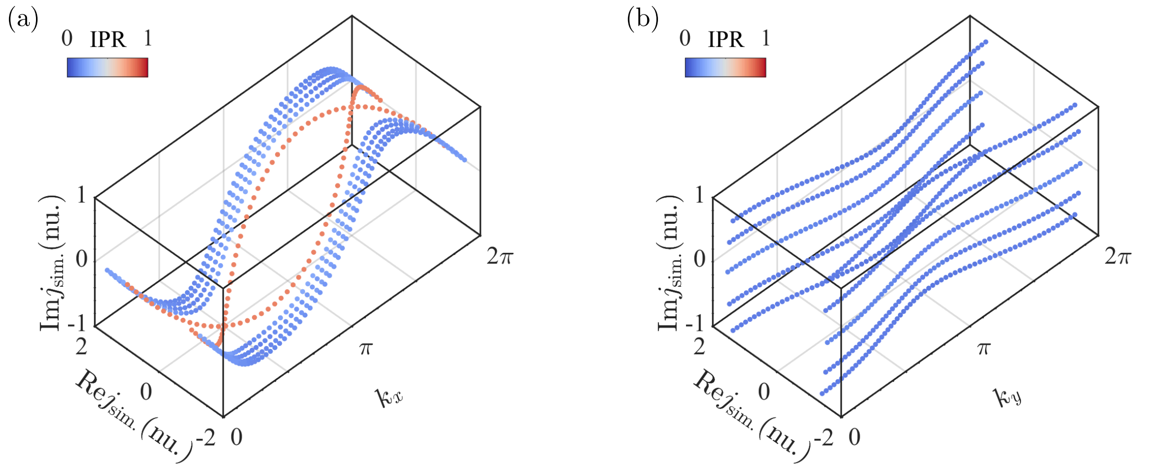

FIG. S1. Spectra of the circuit Laplacian (a) as a function of k_x for PBCx-OBCy and (b) as a function of k_y for OBCx-PBCy.

S3. PHYSICS-GRAPH-INFORMED MACHINE LEARNING

Experimental determination of the S-parameters is time-consuming for a large circuit. Physics-graph-informed machine learning (PGIML) enables accurate estimation of the experimental spectra with a reasonable number of measurements. The algorithm is general, as it does not rely on a specific parametrization of the Hamiltonian, and is readily applicable to any system.

A. From graph to circuit

A graph (circuit) is a mathematical structure to model pairwise relations between vertices (nodes) connected by edges (positions). A circuit is a collection of interconnected components which obey Kirchhoffs laws. Kirchhoffs voltage law requires the sum of the voltages in any closed loop to be zero and Kirchhoffs current law requires the sum of the currents meeting at a node to be zero. Thus, circuits are characterised by linear relationships among the voltage variables and among the current variables. According to graph theory (network topology), a N-port electrical circuit can be converted into a matrix $\boldsymbol{G} = (\boldsymbol{P}, \boldsymbol{S})$ of complex-weighted directed bipartite graphs $G_{ab} = (P_{ab}, S_{ab})$ with the matrix $\boldsymbol{P}$ of positions $P_{ab} = (a, b)$ and the S-matrix $\boldsymbol{S}$ of scattering-parameters (S-parameters) S_{ab}, where $a, b \in \{1, 2, \dots, N\}$ denotes the ports [12]. We define the set of graphs as $\mathcal{G} = (\mathcal{P}, \mathcal{S}) = \{G_{ab} | a, b \in \{1, 2, \dots, N\}\}$ with the set of positions $\mathcal{P}$ and the set of S-parameters $\mathcal{S}$.

B. K-means method

The K-means method is an unsupervised method to classify data points into K mutually exclusive clusters such that the data points in the same cluster are as similar as possible (i.e., high intra-class similarity) and the data points in different clusters are as dissimilar as possible (i.e., low inter-class similarity) [13, 14]. Each cluster is classified by its centroid (= mean of the values assigned to the cluster). We define the distance between two S-parameters as

$$d(S_i, S_j) = \|S_i - S_j\|_2^2. \tag{S77}$$

Given $\mathcal{G} = (\mathcal{P}, \mathcal{S})$, representing a circuit with N nodes, the K-means method, see Alg. S1, classifies the set $\mathcal{S} = \{S_1, S_2, \ldots, S_{N^2}\}$ of S-parameters (relabel the set of indices $\{(a,b)|a,b \in 1,2,\ldots,N\} \to \{1,2,\ldots,N^2\}$) into clusters $\mathcal{S}_1, \mathcal{S}_2, \ldots, \mathcal{S}_K$ and the set $\mathcal{P} = \{P_1, P_2, \ldots, P_{N^2}\}$ of positions into clusters $\mathcal{P}_1, \mathcal{P}_2, \ldots, \mathcal{P}_K$. Firstly, K centroids are randomly selected from $\mathcal{S}$. Secondly, the distances to all centroids are calculated for every point in $\mathcal{S}$, and the point is assigned to the closest cluster. Thirdly, the centroid is recomputed for every cluster. Steps two and three are repeated until convergence or the maximal number of iterations is reached. Alg. S1 partitions $\mathcal{G}$ into subgraphs $\mathcal{G}_1, \mathcal{G}_2, \ldots, \mathcal{G}_K$ (clusters) based on the S-parameters.

Algorithm S1 K-means

Input
    $\mathcal{S} = \{S_1, S_2, \ldots, S_{N^2}\}$: Set of S-parameters
    K: Number of clusters
Output
    $\{\mathcal{S}_1, \mathcal{S}_2, \ldots, \mathcal{S}_K\}$: Set of clusters of S-parameters
    $\{\mathcal{P}_1, \mathcal{P}_2, \ldots, \mathcal{P}_K\}$: Set of clusters of positions
    $\{C_1, C_2, \ldots, C_K\}$: Set of centroids
Define centroids $C_1, C_2, \ldots, C_K$ by randomly selecting K points from $\mathcal{S}$
while Convergence and maximal number of iterations are not reached **do**
    for $n = 1$ to N^2 **do**
        $\kappa = \arg\min_n \|S_n - C_\kappa\|_2^2$
        $(\tilde{\mathcal{P}}_\kappa, \tilde{\mathcal{S}}_\kappa)$.append$(P_n, S_n)$
    for $\kappa = 1$ to K **do**
        $C_\kappa = \text{mean}(\tilde{\mathcal{S}}_\kappa)$
        $(\mathcal{P}_\kappa, \mathcal{S}_\kappa) = (\tilde{\mathcal{P}}_\kappa, \tilde{\mathcal{S}}_\kappa)$
        $(\tilde{\mathcal{P}}_\kappa, \tilde{\mathcal{S}}_\kappa) = (\emptyset, \emptyset)$
return $\{\mathcal{S}_1, \mathcal{S}_2, \ldots, \mathcal{S}_K\}$, $\{\mathcal{P}_1, \mathcal{P}_2, \ldots, \mathcal{P}_K\}$, and $\{C_1, C_2, \ldots, C_K\}$

C. Reconstruction

We classify the set of simulated S-parameters $\mathcal{S}_{\text{sim.}} = \{S_{\text{sim.},1}, S_{\text{sim.},2}, \ldots, S_{\text{sim.},N^2}\}$ into clusters $\mathcal{S}_{\text{sim.},1}$, $\mathcal{S}_{\text{sim.},2}$, $\ldots, \mathcal{S}_{\text{sim.},K}$ and obtain for every cluster $\mathcal{S}_{\text{sim.},\kappa}$ the centroid $C_{\text{sim.},\kappa}$ which may not belong to $\mathcal{S}_{\text{sim.}}$. The set of simulated graphs $\mathcal{G}_{\text{sim.}}$ is an approximation of the set of experimental graphs $\mathcal{G}_{\text{exp.}}$. As $\mathcal{G}_{\text{sim.}}$, $\mathcal{G}_{\text{exp.}}$ are isomorphic and $\mathcal{G}_{\text{sim.},\kappa}$ and $\mathcal{G}_{\text{exp.},\kappa}$ are isomorphic. Therefore, $\mathcal{G}_{\text{sim.}}$ provides informative priors to $\mathcal{G}_{\text{exp.}}$ by the mapping $\hat{G}_{\text{sim.},\kappa} \to \hat{G}_{\text{exp.},\kappa}$, where $\hat{G}_{\text{sim.}} = (\hat{P}_{\text{sim.}}, \hat{S}_{\text{sim.}})$ and $\hat{G}_{\text{exp.}} = (\hat{P}_{\text{exp.}}, \hat{S}_{\text{exp.}})$ are representative graphs of $\mathcal{G}_{\text{sim.}}$ and $\mathcal{G}_{\text{exp.}}$, respectively. By encoding the S-matrix with the representative experimental S-parameters $\hat{S}_{\text{exp.},1}, \hat{S}_{\text{exp.},2}, \ldots, \hat{S}_{\text{exp.},\kappa}$, we obtain the reconstructed experimental S-matrix $\hat{\boldsymbol{S}}_{\text{exp.}}$, see Alg. S2.

Algorithm S2 Reconstruction

Input
  $\mathcal{S}_{\text{sim.}} = \{S_{\text{sim.},1}, S_{\text{sim.},2}, \ldots, S_{\text{sim.},N^2}\}$: Set of simulated S-parameters
  K: Number of clusters
Output
  $\hat{\boldsymbol{S}}_{\text{exp.}}$: Reconstructed experimental S-matrix
Classify $\mathcal{S}_{\text{sim.}}$ into clusters $\mathcal{S}_{\text{sim.},1}, \mathcal{S}_{\text{sim.},2}, \ldots, \mathcal{S}_{\text{sim.},K}$ and $\mathcal{P}_{\text{sim.}}$ into clusters $\mathcal{P}_{\text{sim.},1}, \mathcal{P}_{\text{sim.},2}, \ldots, \mathcal{P}_{\text{sim.},K}$ with centroids $C_{\text{sim.},1}, C_{\text{sim.},2}, \ldots, C_{\text{sim.},K}$ by Alg. S1
for $\kappa = 1$ to K **do**
  $\hat{S}_{\text{sim.},\kappa} = \arg\min_{S \in \mathcal{S}_{\text{sim.},\kappa}} \|S - C_{\text{sim.},\kappa}\|_2^2$
  $\hat{S}_{\text{sim.},\kappa} \to \hat{P}_{\text{sim.},\kappa} \to (a, b)$
  Measure $\hat{S}_{\text{exp.},\kappa}$ between port a and b
  for $n = 1$ to N^2 **do**
    if $n \in \mathcal{P}_{\text{sim.},\kappa}$ **then**
      $n \to (a, b)$
      $\hat{\boldsymbol{S}}_{\text{exp.}} = \hat{\boldsymbol{S}}_{\text{exp.}} + \hat{S}_{\text{exp.},\kappa} \boldsymbol{E}_{ab}$
return $\hat{\boldsymbol{S}}_{\text{exp.}}$

D. Execution of the PGIML method

We consider a 10×10 lattice and Eq. (S23) with the parameters below Eq. (S24). Fig. S2(a) shows the simulated J-matrix $\boldsymbol{J}_{\text{sim.}}$, which can be transformed into the simulated S-matrix $\boldsymbol{S}_{\text{sim.}}$, see Sec. S1 B. To check the validity of the PGIML method, we compare $\boldsymbol{S}_{\text{sim.}}$ with the reconstructed simulated S-matrix $\hat{\boldsymbol{S}}_{\text{sim.}}$. The K-means method is used to classify $\mathcal{S}_{\text{sim.}}$ into $K = 40$ clusters, see Fig. S2(b). For the κ-th cluster with centroid $C_{\text{sim.},\kappa}$, we select $\hat{P}_{\text{sim.},\kappa}$ such that $\hat{S}_{\text{sim.},\kappa} =$

$\arg\min_{S\in\mathcal{S}_{\text{sim.},\kappa}} \|S - C_{\text{sim.},\kappa}\|_2^2$. By encoding the S-matrix with $\hat{S}_{\text{sim.},1}, \hat{S}_{\text{sim.},2}, \ldots, \hat{S}_{\text{sim.},\kappa}$, see Fig. S2(c), we obtain $\hat{\boldsymbol{S}}_{\text{sim.}}$, which can be transformed back into the reconstructed simulated J-matrix $\hat{\boldsymbol{J}}_{\text{sim.}}$, see Fig. S2(d).

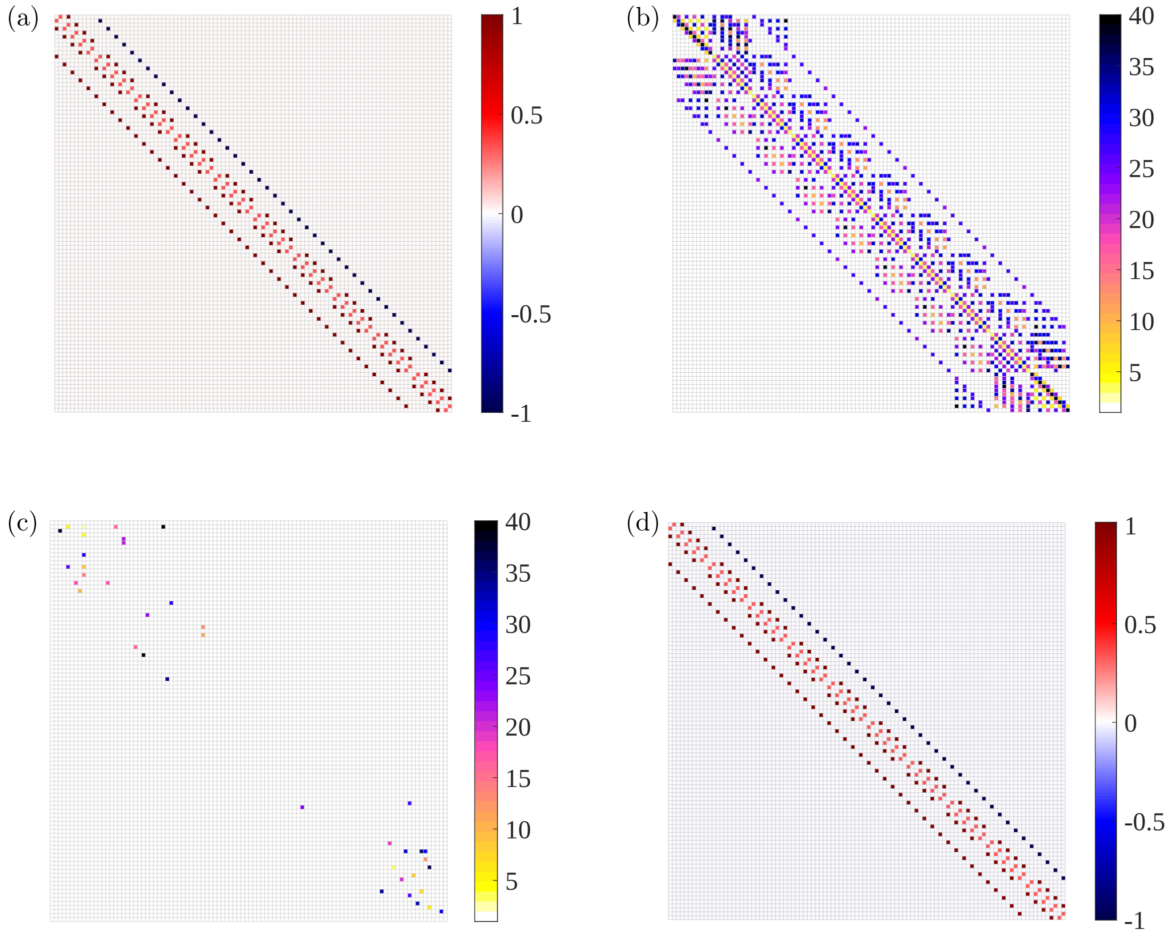

FIG. S2. (a) Simulated J-matrix, (b) clusters of the simulated S-parameters, (c) representative simulated S-parameters, and (d) reconstructed simulated J-matrix.

To evaluate the accuracy of the algorithm, we calculate the mean squared error (MSE) as loss function,

$$\mathrm{MSE} = \sum_{n=1}^{N^2} \frac{\|\hat{S}_{\mathrm{sim.,n}} - S_{\mathrm{sim.,n}}\|_2^2}{N^2}, \tag{S78}$$

which measures the compactness of the clustering and should be as small as possible. Figure S3 shows the MSE versus the number of clusters for different boundary conditions. When the MSE is smaller than 10^{-9}, we assume $\hat{\boldsymbol{J}}_{\mathrm{sim.}}$ to be sufficiently similar to $\boldsymbol{J}_{\mathrm{sim.}}$, i.e., K is large enough to determine the S-matrix (underfitting and wellfitting regimes in Fig. S3). We find a larger MSE for OBCx-OBCy, i.e., we have to choose a larger K than for the other boundary conditions. The spectra $j_{\mathrm{sim.}}$ of $\boldsymbol{J}_{\mathrm{sim.}}$ and $\hat{j}_{\mathrm{sim.}}$ of $\hat{\boldsymbol{J}}_{\mathrm{sim.}}$ for the cases of underfitting and wellfitting are compared in Fig. S4 for different boundary conditions. In the case of underfitting, we observe a poor predictive performance, while in the case of wellfitting the reconstructed results resemble the simulated results. Our estimator also provides remarkable accuracy for a large system, such as a 30×30 lattice, but a larger number of clusters is required, see Fig. S5.

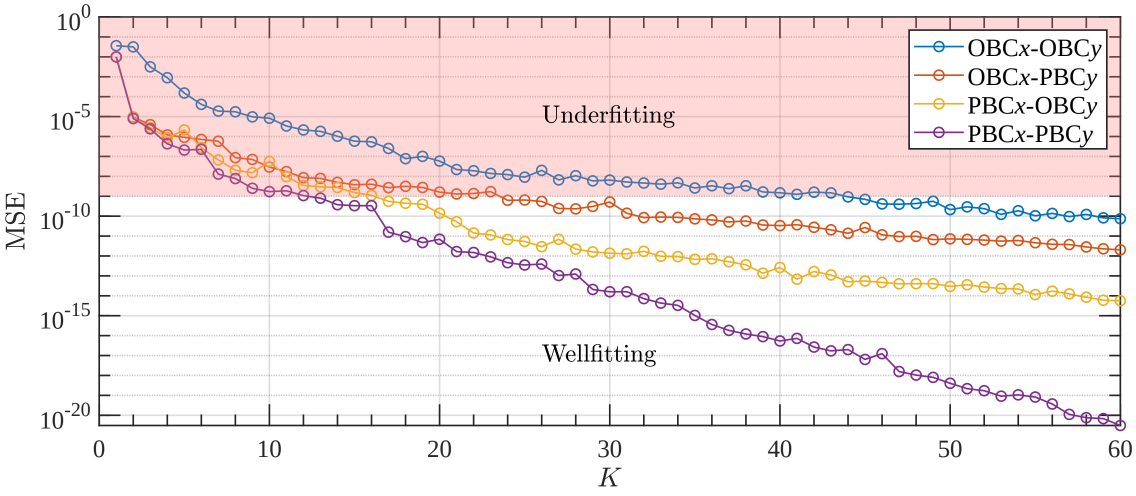

FIG. S3. MSE versus the number of clusters for different boundary conditions.

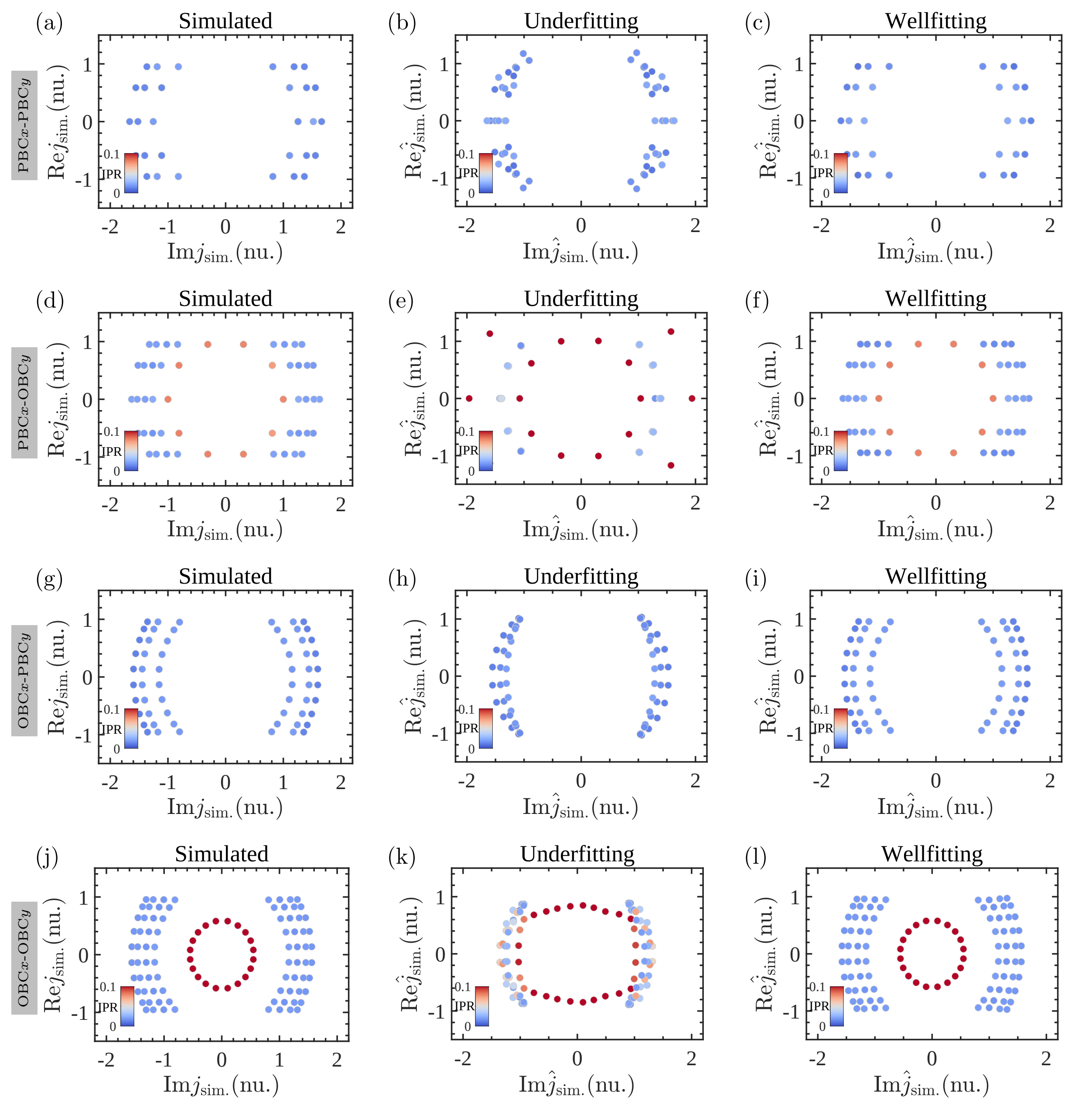

FIG. S4. Spectra of the J-matrix on a 10×10 lattice for different boundary conditions: (a, d, g, j) simulated, (b, e, h, k) reconstructed with $K = 5$ (underfitting), and (c, f, i, l) reconstructed with $K = 40$ (wellfitting).

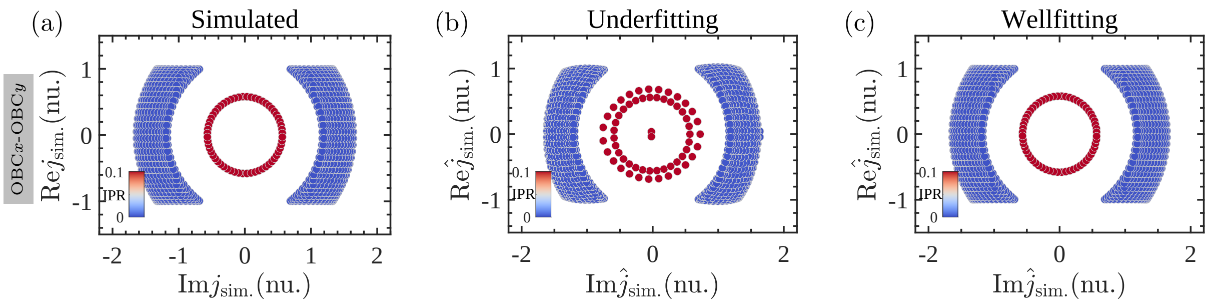

FIG. S5. Spectra of the J-matrix on a 30×30 lattice for OBCx-OBCy: (a) simulated, (b) reconstructed with $K = 80$ (underfitting), and (c) reconstructed with $K = 250$ (wellfitting).

S4. EXPERIMENTAL IMPLEMENTATION

We introduce the experimental setup, fitting model, and selection of measurements.

A. Experimental setup

To map the lattice Hamiltonian to a circuit Laplacian, we eliminate the diagonal elements of the circuit Laplacian at the resonance frequency by choosing appropriate inductivities and capacitances and canceling the contributions of the inductivities (capacitances) at the nodes by grounding components with matched capacitances (inductivities). As shown in Fig. S6, we divide the circuit into corner (NW, SW, NE, SE), edge ($\rm N_A$, $\rm S_B$, $\rm W_A$, $\rm W_B$, $\rm E_A$, $\rm E_B$), and bulk ($\rm M_A$, $\rm M_B$) sites (N = north, S = south, W = west, E = east, M = middle). The unit cell of the circuit is shown in Fig. S7(a). The boundary conditions are realized by adjusting the switches to the ground and connections between the boundaries. For instance, to create PBCx, we turn on the switches $T_{\rm W\to S}$ and turn off the switch $T_{\rm W_A}$. We present the grounding components for the different boundary conditions in Table S1. The circuit sites are connected by pairs of inductors (L_1, L_2), pairs of capacitors (C_1, C_2), and voltage feedback operational amplifiers (nonreciprocal couplings; Texas Instruments, LM6171), as shown in Fig. S7(b), which block the input current while maintaining the output current. The inductors and capacitors are Hermitian components while the resistors and voltage feedback operational amplifiers are non-Hermitian components, see Fig. S7(c).

	OBCx-OBCy	OBCx-PBCy	PBCx-OBCy	PBCx-PBCy
NW	$2C_1 \parallel L_1/2 \parallel L_2$	$C_1 \parallel L_1/2 \parallel L_2$	$C_1 \parallel L_1/2 \parallel L_2$	$L_1/2 \parallel L_2$
SW	$C_1 \parallel L_2$	L_2	$C_1 \parallel L_2$	L_2
NE	$C_1 \parallel L_1/2 \parallel L_2$	$L_1/2 \parallel L_2$	$C_1 \parallel L_1/2 \parallel L_2$	$L_1/2 \parallel L_2$
SE	$C_1 \parallel L_1 \parallel L_2$	$L_1 \parallel L_2$	$C_1 \parallel L_2$	L_2
$\mathrm{N_A}$	$C_1 \parallel L_1/2 \parallel L_2$	$L_1/2 \parallel L_2$	$C_1 \parallel L_1/2 \parallel L_2$	$L_1/2 \parallel L_2$
$\mathrm{S_B}$	$C_1 \parallel L_2$	L_2	$C_1 \parallel L_2$	L_2
$\mathrm{W_A}$	$C_1 \parallel L_1/2 \parallel L_2$	$C_1 \parallel L_1/2 \parallel L_2$	$L_1/2 \parallel L_2$	$L_1/2 \parallel L_2$
$\mathrm{W_B}$	L_2	L_2	L_2	L_2
$\mathrm{E_A}$	$L_1/2 \parallel L_2$	$L_1/2 \parallel L_2$	$L_1/2 \parallel L_2$	$L_1/2 \parallel L_2$
$\mathrm{E_B}$	$L_1 \parallel L_2$	$L_1 \parallel L_2$	L_2	L_2
$\mathrm{M_A}$	$L_1/2 \parallel L_2$	$L_1/2 \parallel L_2$	$L_1/2 \parallel L_2$	$L_1/2 \parallel L_2$
$\mathrm{M_B}$	L_2	L_2	L_2	L_2

TABLE S1. Grounding components for different boundary conditions, where $\parallel$ indicates parallel connection.

B. Fitting model

To take into account the parasitic resistances R_{L_j} of the inductors as the most prominent experimental imperfections, we adjust the inductances as $L_j^{'} = L_j + R_{L_j}/(\mathrm{i}\omega), j = 1, 2$, i.e., Eq. (S23) is rewritten as

$$\boldsymbol{J}_{\text{fit.}}(\boldsymbol{k}, \omega) = \mathrm{i}\omega \begin{bmatrix} \frac{L_1^{'}L_2^{'}}{(L_1^{'}+2L_2^{'})\omega^2} - 2C_1 - C_2 + C_1 e^{-\mathrm{i}k_x} & C_2 + C_1 e^{-\mathrm{i}k_y} \\ C_2 + C_1 e^{\mathrm{i}k_y} & \frac{L_1^{'}L_2^{'}}{(L_1^{'}+L_2^{'})\omega^2} - C_1 - C_2 - \frac{L_1^{'}}{\omega^2} e^{\mathrm{i}k_x} \end{bmatrix}. \tag{S79}$$

For $R_{L_j} = 7\,\Omega$, we obtain the spectra $j_{\text{fit.}}$ of the fitted J-matrix in Fig. S8 for different boundary conditions. Compared with Figs. S4(a, d, g, j), the spectra in Figs. S8(a, b) show deviations in terms of non-degenerate modes due to the parasitic resistances, as observed in the experiments, while the spectra in Figs. S8(c, d) show no such deviations due to the fact that there are no degenerate modes.

C. Selection of measurements

We choose $K = 100$, i.e., more than the $K = 40$ clusters considered in Sec. S3 D due to the imperfections of the components and instability of the voltage feedback operational amplifiers.

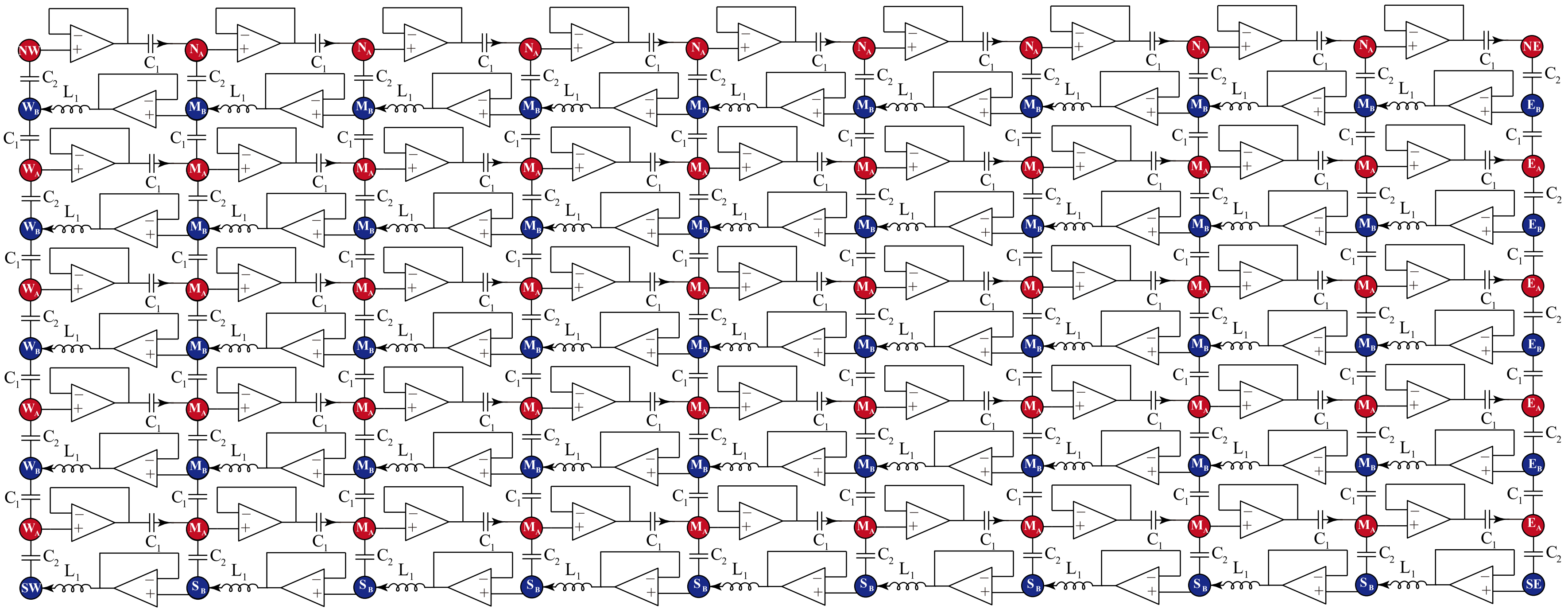

FIG. S6. Circuit implementation of the second order NHSE.

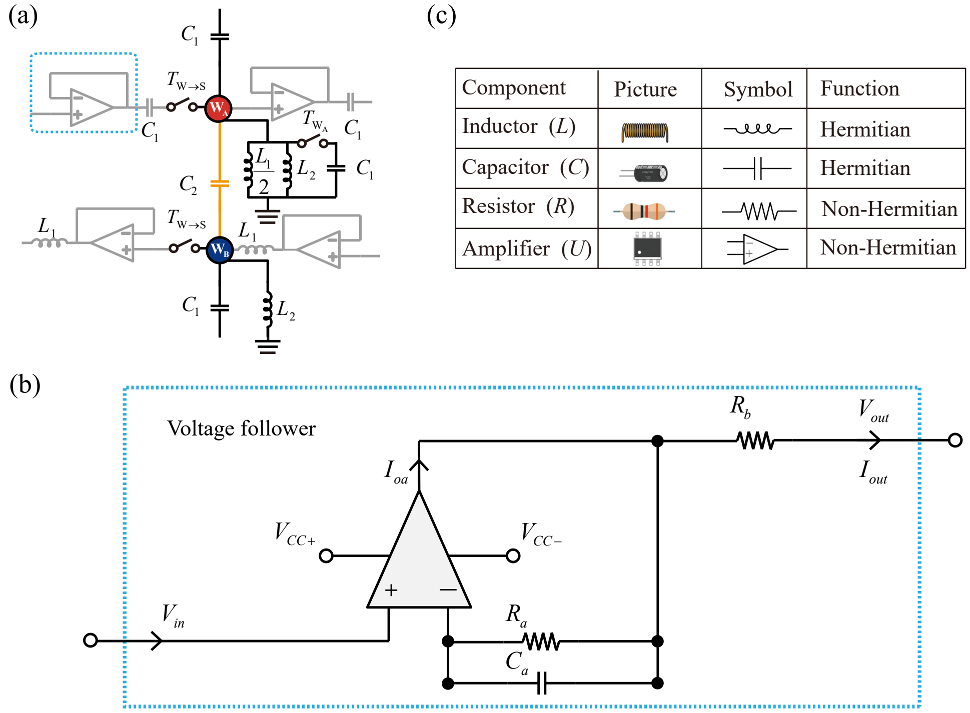

FIG. S7. (a) Unit cell at the boundary of the circuit and switches to control the boundary condition, (b) voltage follower, and (c) functions of the components.

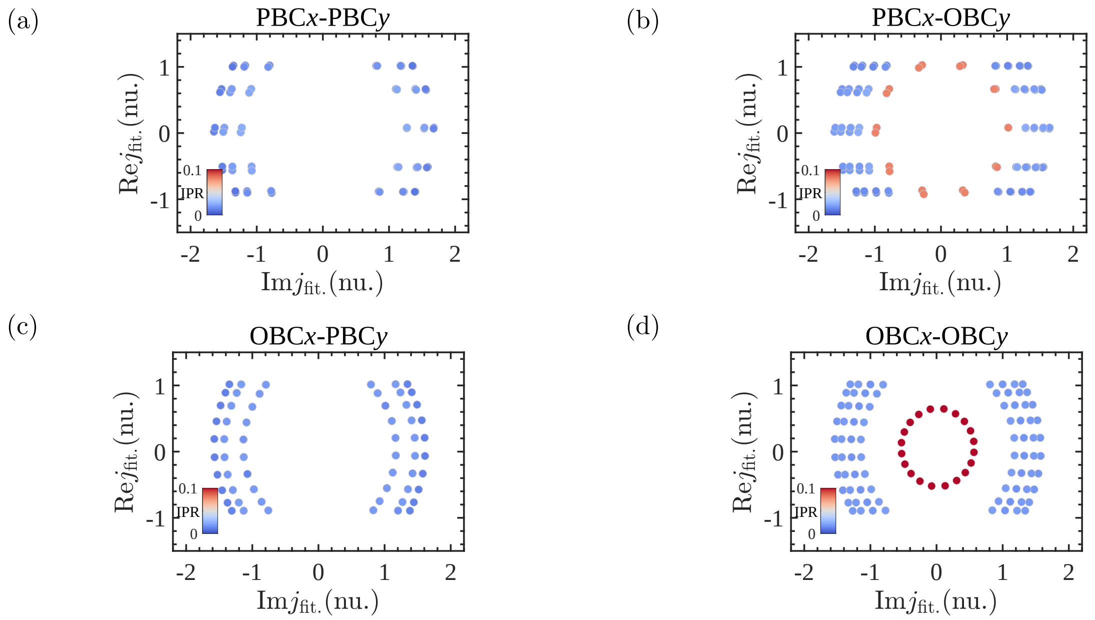

FIG. S8. Spectra of the fitted J-matrix for (a) PBCx-PBCy, (b) PBCx-OBCy, (c) OBCx-PBCy, and (d) OBCx-OBCy.

More clusters provide not only more accuracy of the reconstruction according to Fig. S3 but also offer better correction of the components. The selected sites for the measurements are presented in Table S2. For example, the measurement for cluster $\kappa = 1$ for PBCx-PBCy ($\hat{S}_{\text{exp.},1}$) is executed between sites $(0,0)$ and $(2,2)$, denoted as $(0,0) \to (2,2)$. As shown in Fig. S9, every site is measured at least once. If the measured result agrees with the simulated result, all grounding and connecting components operate correctly; if not, the welding must be checked.

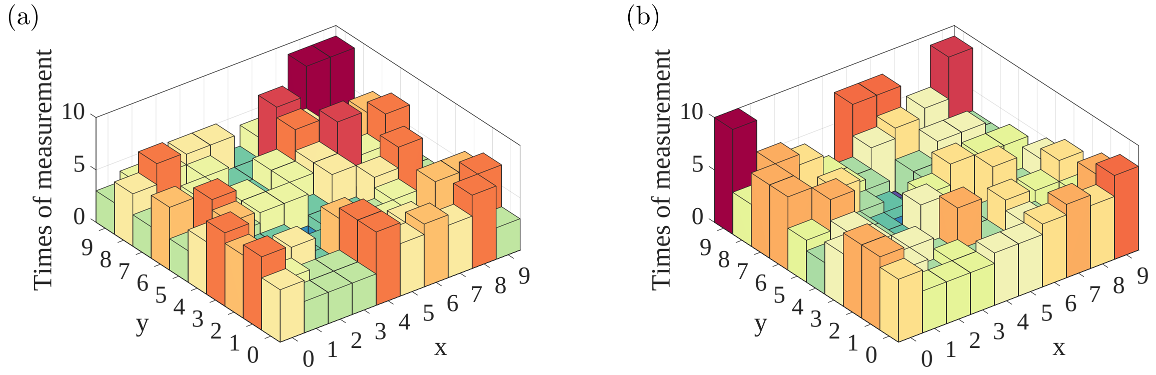

FIG. S9. Statistics of the measurements for S_{ab} between (a) site a and (b) site b. The sites are labeled $0, 1, \ldots, 9$ along the x and y-directions.

κ	PBCx-PBCy	PBCx-OBCy	OBCx-PBCy	OBCx-OBCy
1	$(0,0) \to (2,2)$	$(0,0) \to (0,0)$	$(0,0) \to (0,0)$	$(0,0) \to (0,0)$
2	$(0,3) \to (0,3)$	$(0,1) \to (3,2)$	$(0,1) \to (0,1)$	$(0,0) \to (1,0)$
3	$(0,3) \to (3,2)$	$(0,2) \to (0,2)$	$(0,1) \to (0,2)$	$(0,1) \to (0,0)$
4	$(0,4) \to (1,3)$	$(0,3) \to (1,0)$	$(0,1) \to (1,2)$	$(0,1) \to (0,2)$
5	$(0,5) \to (7,5)$	$(0,3) \to (9,1)$	$(0,2) \to (0,2)$	$(0,1) \to (0,3)$
6	$(0,6) \to (9,7)$	$(0,4) \to (0,4)$	$(0,2) \to (0,3)$	$(0,2) \to (0,4)$
7	$(0,7) \to (1,7)$	$(0,5) \to (0,5)$	$(0,2) \to (1,1)$	$(0,3) \to (0,2)$
8	$(0,7) \to (9,9)$	$(0,6) \to (5,2)$	$(0,2) \to (1,2)$	$(0,4) \to (0,3)$
9	$(0,9) \to (0,9)$	$(0,8) \to (1,5)$	$(0,3) \to (0,1)$	$(0,6) \to (0,6)$
10	$(1,1) \to (2,8)$	$(0,9) \to (0,9)$	$(0,3) \to (0,2)$	$(0,6) \to (0,7)$
11	$(1,2) \to (0,5)$	$(1,1) \to (1,1)$	$(0,4) \to (1,5)$	$(0,6) \to (2,6)$
12	$(1,4) \to (0,6)$	$(1,1) \to (9,2)$	$(0,4) \to (2,2)$	$(0,7) \to (0,7)$
13	$(1,4) \to (1,6)$	$(1,3) \to (1,1)$	$(0,5) \to (1,4)$	$(0,8) \to (0,9)$
14	$(1,4) \to (2,4)$	$(1,4) \to (0,1)$	$(0,6) \to (2,6)$	$(0,8) \to (1,8)$
15	$(1,5) \to (0,6)$	$(1,5) \to (0,5)$	$(0,8) \to (0,6)$	$(0,9) \to (0,9)$
16	$(1,5) \to (9,9)$	$(1,5) \to (2,4)$	$(0,8) \to (0,7)$	$(1,0) \to (1,0)$
17	$(1,8) \to (0,8)$	$(1,7) \to (0,8)$	$(1,0) \to (0,9)$	$(1,0) \to (2,0)$
18	$(2,0) \to (2,0)$	$(1,7) \to (8,7)$	$(1,2) \to (2,2)$	$(1,1) \to (0,1)$
19	$(2,2) \to (0,1)$	$(1,8) \to (0,8)$	$(1,3) \to (0,4)$	$(1,2) \to (3,3)$
20	$(2,5) \to (2,5)$	$(1,8) \to (1,8)$	$(1,4) \to (1,4)$	$(1,4) \to (1,4)$
21	$(2,9) \to (9,9)$	$(1,8) \to (2,8)$	$(1,5) \to (0,5)$	$(1,5) \to (0,6)$
22	$(3,1) \to (2,1)$	$(1,9) \to (1,9)$	$(1,6) \to (0,7)$	$(1,5) \to (2,5)$
23	$(3,3) \to (4,1)$	$(2,2) \to (3,2)$	$(1,9) \to (0,8)$	$(1,6) \to (1,8)$
24	$(3,5) \to (1,5)$	$(2,3) \to (3,1)$	$(2,0) \to (1,3)$	$(1,8) \to (0,7)$
25	$(3,6) \to (2,4)$	$(2,5) \to (5,1)$	$(2,2) \to (2,0)$	$(1,8) \to (2,8)$
26	$(3,7) \to (5,8)$	$(2,7) \to (0,9)$	$(2,2) \to (2,2)$	$(1,8) \to (4,8)$
27	$(3,8) \to (0,6)$	$(2,7) \to (3,6)$	$(2,5) \to (3,5)$	$(1,9) \to (0,9)$
28	$(3,8) \to (3,8)$	$(2,9) \to (1,9)$	$(2,6) \to (3,6)$	$(1,9) \to (2,9)$
29	$(3,9) \to (2,1)$	$(2,9) \to (2,5)$	$(2,7) \to (0,7)$	$(2,0) \to (3,0)$
30	$(3,9) \to (3,9)$	$(3,1) \to (4,2)$	$(2,7) \to (4,9)$	$(2,1) \to (0,3)$
31	$(4,1) \to (4,0)$	$(3,2) \to (3,0)$	$(2,8) \to (1,0)$	$(2,1) \to (1,3)$
32	$(4,1) \to (5,3)$	$(3,5) \to (1,5)$	$(2,8) \to (2,8)$	$(2,1) \to (2,3)$
33	$(4,1) \to (7,2)$	$(3,8) \to (4,7)$	$(3,0) \to (3,0)$	$(2,2) \to (1,4)$
34	$(4,4) \to (4,4)$	$(3,9) \to (1,8)$	$(3,2) \to (1,2)$	$(2,4) \to (1,5)$
35	$(4,5) \to (3,8)$	$(4,0) \to (2,0)$	$(3,5) \to (3,5)$	$(2,4) \to (3,4)$

36	$(4,5) \to (6,5)$	$(4,1) \to (3,1)$	$(3,6) \to (3,6)$	$(2,6) \to (2,6)$
37	$(4,5) \to (8,8)$	$(4,1) \to (6,1)$	$(3,6) \to (4,6)$	$(2,9) \to (0,9)$
38	$(4,6) \to (1,5)$	$(4,2) \to (4,4)$	$(3,6) \to (6,6)$	$(3,0) \to (3,0)$
39	$(4,9) \to (1,8)$	$(4,2) \to (6,2)$	$(3,8) \to (4,0)$	$(3,0) \to (4,0)$
40	$(4,9) \to (4,2)$	$(4,3) \to (3,3)$	$(3,9) \to (2,9)$	$(3,1) \to (1,1)$
41	$(5,1) \to (3,1)$	$(4,5) \to (2,6)$	$(4,0) \to (4,0)$	$(3,4) \to (3,4)$
42	$(5,1) \to (4,1)$	$(4,6) \to (6,5)$	$(4,0) \to (4,4)$	$(3,4) \to (4,4)$
43	$(5,1) \to (4,2)$	$(4,7) \to (3,7)$	$(4,0) \to (5,0)$	$(3,5) \to (2,6)$
44	$(5,3) \to (6,5)$	$(4,8) \to (4,8)$	$(4,0) \to (5,9)$	$(3,9) \to (2,9)$
45	$(5,4) \to (6,3)$	$(4,9) \to (3,9)$	$(4,1) \to (2,1)$	$(4,0) \to (4,0)$
46	$(5,5) \to (3,7)$	$(4,9) \to (6,9)$	$(4,2) \to (4,2)$	$(4,0) \to (5,0)$
47	$(5,5) \to (4,5)$	$(5,0) \to (7,0)$	$(4,2) \to (6,2)$	$(4,1) \to (4,1)$
48	$(5,6) \to (7,3)$	$(5,2) \to (5,2)$	$(4,2) \to (7,1)$	$(4,2) \to (4,2)$
49	$(5,7) \to (3,7)$	$(5,2) \to (6,2)$	$(4,3) \to (4,4)$	$(4,8) \to (5,8)$
50	$(5,7) \to (5,9)$	$(5,6) \to (4,7)$	$(4,7) \to (5,5)$	$(5,0) \to (5,0)$
51	$(5,9) \to (3,8)$	$(5,6) \to (7,5)$	$(4,9) \to (2,8)$	$(5,0) \to (6,0)$
52	$(6,1) \to (6,1)$	$(5,7) \to (7,5)$	$(5,0) \to (5,0)$	$(5,2) \to (6,1)$
53	$(6,2) \to (2,3)$	$(6,0) \to (5,0)$	$(5,0) \to (6,1)$	$(5,8) \to (5,8)$
54	$(6,4) \to (7,4)$	$(6,0) \to (5,1)$	$(5,1) \to (2,2)$	$(5,8) \to (6,8)$
55	$(6,5) \to (5,3)$	$(6,0) \to (7,0)$	$(5,4) \to (6,4)$	$(5,9) \to (5,9)$
56	$(6,5) \to (5,5)$	$(6,2) \to (8,0)$	$(5,6) \to (5,6)$	$(6,0) \to (6,0)$
57	$(6,6) \to (6,0)$	$(6,3) \to (4,2)$	$(5,7) \to (4,6)$	$(6,0) \to (7,0)$
58	$(6,6) \to (8,6)$	$(6,3) \to (5,3)$	$(6,0) \to (7,0)$	$(6,1) \to (5,1)$
59	$(6,7) \to (1,3)$	$(6,3) \to (8,4)$	$(6,1) \to (3,1)$	$(6,1) \to (6,2)$
60	$(6,7) \to (7,7)$	$(6,5) \to (5,5)$	$(6,1) \to (6,0)$	$(6,4) \to (7,4)$
61	$(6,7) \to (9,8)$	$(6,6) \to (0,6)$	$(6,2) \to (5,9)$	$(6,5) \to (6,4)$
62	$(6,8) \to (5,5)$	$(6,6) \to (5,4)$	$(6,2) \to (8,3)$	$(6,6) \to (6,6)$
63	$(6,8) \to (6,7)$	$(6,7) \to (3,8)$	$(6,5) \to (6,2)$	$(6,8) \to (8,8)$
64	$(6,8) \to (7,8)$	$(6,7) \to (6,8)$	$(6,7) \to (6,0)$	$(6,9) \to (6,8)$
65	$(6,9) \to (4,7)$	$(6,7) \to (6,9)$	$(6,8) \to (6,8)$	$(7,0) \to (6,0)$
66	$(7,1) \to (6,1)$	$(6,8) \to (6,5)$	$(7,0) \to (8,9)$	$(7,0) \to (7,0)$
67	$(7,3) \to (9,2)$	$(6,8) \to (6,9)$	$(7,2) \to (9,2)$	$(7,0) \to (8,0)$
68	$(7,3) \to (9,3)$	$(6,8) \to (8,9)$	$(7,6) \to (5,4)$	$(7,0) \to (8,2)$
69	$(7,4) \to (7,4)$	$(6,9) \to (2,9)$	$(7,6) \to (8,3)$	$(7,2) \to (7,2)$
70	$(7,4) \to (8,2)$	$(6,9) \to (5,9)$	$(7,6) \to (8,6)$	$(7,3) \to (7,0)$
71	$(7,6) \to (1,6)$	$(7,3) \to (6,3)$	$(7,7) \to (5,8)$	$(7,4) \to (7,1)$
72	$(7,6) \to (1,7)$	$(7,4) \to (5,8)$	$(7,8) \to (6,8)$	$(7,4) \to (8,4)$

73	$(7,6) \to (8,7)$	$(7,5) \to (6,5)$	$(7,8) \to (7,8)$	$(7,5) \to (8,3)$
74	$(7,7) \to (3,7)$	$(7,7) \to (6,7)$	$(7,9) \to (9,0)$	$(7,5) \to (9,6)$
75	$(7,7) \to (7,4)$	$(7,7) \to (7,6)$	$(8,0) \to (8,0)$	$(7,6) \to (6,6)$
76	$(7,9) \to (6,9)$	$(7,8) \to (1,8)$	$(8,0) \to (9,0)$	$(7,6) \to (8,7)$
77	$(8,2) \to (0,1)$	$(7,8) \to (4,8)$	$(8,1) \to (8,9)$	$(7,8) \to (5,9)$
78	$(8,2) \to (6,2)$	$(8,0) \to (1,1)$	$(8,1) \to (9,0)$	$(8,0) \to (8,0)$
79	$(8,2) \to (7,4)$	$(8,0) \to (2,8)$	$(8,2) \to (9,3)$	$(8,0) \to (9,0)$
80	$(8,2) \to (9,9)$	$(8,0) \to (8,0)$	$(8,3) \to (9,4)$	$(8,2) \to (9,3)$
81	$(8,3) \to (6,5)$	$(8,1) \to (9,1)$	$(8,4) \to (9,3)$	$(8,4) \to (6,3)$
82	$(8,4) \to (4,2)$	$(8,7) \to (6,7)$	$(8,5) \to (7,7)$	$(8,4) \to (9,3)$
83	$(8,4) \to (8,1)$	$(8,8) \to (5,9)$	$(8,6) \to (9,9)$	$(8,4) \to (9,4)$
84	$(8,4) \to (8,5)$	$(8,8) \to (9,9)$	$(8,8) \to (7,7)$	$(8,6) \to (8,6)$
85	$(8,5) \to (1,4)$	$(8,9) \to (6,9)$	$(8,9) \to (0,1)$	$(8,9) \to (1,8)$
86	$(8,5) \to (9,3)$	$(8,9) \to (7,9)$	$(8,9) \to (8,0)$	$(8,9) \to (5,9)$
87	$(8,6) \to (0,7)$	$(9,0) \to (0,0)$	$(9,1) \to (9,0)$	$(9,0) \to (9,0)$
88	$(8,6) \to (1,6)$	$(9,2) \to (7,2)$	$(9,1) \to (9,1)$	$(9,0) \to (9,1)$
89	$(8,7) \to (6,9)$	$(9,2) \to (9,1)$	$(9,1) \to (9,9)$	$(9,1) \to (9,1)$
90	$(8,9) \to (6,9)$	$(9,4) \to (0,2)$	$(9,2) \to (8,1)$	$(9,1) \to (9,2)$
91	$(8,9) \to (7,9)$	$(9,5) \to (1,5)$	$(9,2) \to (9,0)$	$(9,5) \to (8,5)$
92	$(8,9) \to (9,6)$	$(9,6) \to (2,6)$	$(9,2) \to (9,1)$	$(9,5) \to (9,4)$
93	$(9,1) \to (1,3)$	$(9,6) \to (7,4)$	$(9,3) \to (8,3)$	$(9,6) \to (9,6)$
94	$(9,1) \to (5,2)$	$(9,6) \to (8,9)$	$(9,3) \to (8,4)$	$(9,6) \to (9,7)$
95	$(9,2) \to (7,0)$	$(9,6) \to (9,6)$	$(9,4) \to (9,4)$	$(9,7) \to (7,7)$
96	$(9,3) \to (7,1)$	$(9,7) \to (6,8)$	$(9,4) \to (9,5)$	$(9,7) \to (8,6)$
97	$(9,7) \to (0,0)$	$(9,7) \to (9,4)$	$(9,6) \to (8,7)$	$(9,8) \to (9,7)$
98	$(9,8) \to (9,8)$	$(9,8) \to (8,7)$	$(9,7) \to (7,7)$	$(9,9) \to (8,9)$
99	$(9,9) \to (0,9)$	$(9,9) \to (0,9)$	$(9,9) \to (8,8)$	$(9,9) \to (9,8)$
100	$(9,9) \to (9,9)$	$(9,9) \to (6,9)$	$(9,9) \to (9,0)$	$(9,9) \to (9,9)$

TABLE S2: Sites selected for the measurements.

---

[1] C. H. Lee, S. Imhof, C. Berger, F. Bayer, J. Brehm, L. W. Molenkamp, T. Kiessling, and R. Thomale, Topolectrical circuits, Commun. Phys. **1**, 39 (2018).

[2] S. Imhof, C. Berger, F. Bayer, J. Brehm, L. W. Molenkamp, T. Kiessling, F. Schindler, C. H. Lee, M. Greiter, T. Neupert, and R. Thomale, Topolectrical-circuit realization of topological corner modes, Nat. Phys. **14**, 925 (2018).

[3] C. G. Montgomery, R. H. Dicke, E. M. Purcell, and E. M. Purcell, *Principles of microwave circuits* (Institute of Electrical Engineers, London, 1987).

[4] D. M. Pozar, *Microwave engineering* (John Wiley & Sons, Hoboken, 2011).

[5] A. Alase, E. Cobanera, G. Ortiz, and L. Viola, Exact solution of quadratic Fermionic Hamiltonians for arbitrary boundary conditions, Phys. Rev. Lett. **117**, 076804 (2016).

[6] S. Yao and Z. Wang, Edge states and topological invariants of non-Hermitian systems, Phys. Rev. Lett. **121**, 086803 (2018).

[7] F. Song, S. Yao, and Z. Wang, Non-Hermitian skin effect and chiral damping in open quantum systems, Phys. Rev. Lett. **123**, 170401 (2019).

[8] K. Yokomizo and S. Murakami, Non-Bloch band theory of non-Hermitian systems, Phys. Rev. Lett. **123**, 066404 (2019).

[9] K. Zhang, Z. Yang, and C. Fang, Correspondence between winding numbers and skin modes in non-Hermitian systems, Phys. Rev. Lett. **125**, 126402 (2020).

[10] Z. Yang, K. Zhang, C. Fang, and J. Hu, Non-Hermitian bulk-boundary correspondence and auxiliary generalized Brillouin zone theory, Phys. Rev. Lett. **125**, 226402 (2020).

[11] M. König, H. Buhmann, L. W. Molenkamp, T. Hughes, C.-X. Liu, X.-L. Qi, and S.-C. Zhang, The quantum spin Hall effect: Theory and experiment, J. Phys. Soc. Jpn. **77**, 031007 (2008).

[12] D. B. West, *Introduction to graph theory* (Prentice Hall, Hoboken, 2001).

[13] K. Krishna and M. N. Murty, Genetic K-means algorithm, IEEE Trans. Syst. Man. **29**, 433 (1999).

[14] A. Likas, N. Vlassis, and J. J. Verbeek, The global K-means clustering algorithm, Pattern Recognit. **36**, 451 (2003).
